# A variational phase-field model for ductile fracture depending on hydrostatic stresses

**DOI:** 10.1007/s11012-025-01971-x

**Published:** 2025-06-25

**Authors:** Anne-Sophie Sur, Laura De Lorenzis, Corrado Maurini, Odd Sture Hopperstad

**Affiliations:** 1 Department of Structural Engineering, Norwegian University of Science and Technology (NTNU), Richard Birkelands vei 1A, 7034 Trondheim, Norway; 2 Department of Mechanical and Process Engineering, ETH Zürich, Tannenstr. 3, 8092 Zürich, Switzerland; 3 Institut Jean Le Rond d’Alembert, Sorbonne University and CNRS, UMR 7190, 75252 Paris, France; 4 Institute of Numerical Mathematics, Technische Universität Dresden (TUD), Zellescher Weg 21-25a, 01069 Dresden, Germany

**Keywords:** Phase-field, Ductile fracture, Stress triaxiality, Variational approach

## Abstract

We model ductile fracture for geometrically linear deformations by coupling plasticity and phase-field fracture models in a variationally consistent framework. The main aim of the proposed model is to account for the effect of stress triaxiality, in order to accurately reproduce ductile fracture, in particular, the instant and location of fracture initiation. For this purpose, we couple the modified Cam-Clay plasticity model with a phase-field fracture model. We study the behaviour of the model analytically in terms of homogeneous material responses, and numerically on plane-strain and axisymmetric specimens under tension with different notches.

## Introduction

Ductile fracture is the failure of materials associated with plastic deformation, and its correct understanding and modelling are important for the safety of engineering structures involving metals, polymers and composites. Complex, interacting, material-specific processes influencing the fracture behaviour, such as nucleation, growth and coalescence of voids, the stress state, and plastic strain, need to be considered to model ductile fracture. Various local damage models have been proposed and extensively used to model ductile fracture in different elasto-plastic materials, including some models which account for stress triaxiality effects [1–8]. However, the localisation of damage results in the loss of ellipticity of the incremental equilibrium equations, which leads to undesirable mesh dependency in finite element (FE) simulations [9–12].

For brittle fracture, phase-field modelling has emerged as an elegant and robust approach to regularise local damage models and retrieve an energetic equivalence with sharp-interface fracture models. The introduction of variational phase-field models to describe fracture goes back to Bourdin et al. [13]; it is based on the variational approach to fracture [14], which formulates brittle fracture mechanics as an energy minimisation problem. In phase-field models a regularisation is introduced by the penalisation of the gradient of the internal variable in the energy functional, involving an internal length. For vanishing internal length, the global minimisers of the regularised models converge to the sharp-interface Griffith models in the sense of $\Gamma$-convergence.

Associative plasticity can also be formulated as an energy minimisation problem [15–17]. Thus, a few models have attempted to couple plasticity and phase-field modelling of fracture within the variational framework. Alessi et al. [18] extend the phase-field approach to ductile fracture by introducing an energy functional describing elasto-plastic material behaviour coupled with a phase-field damage model in a one-dimensional context. Further works by the same authors [19, 20] formulate the model in the multi-dimensional setting for von Mises perfect plasticity, but limit the study of its response to a uniaxial tension test. For the anti-plane case, Dal Maso et al. [21] prove the $\Gamma$-convergence of these models to cohesive fracture models. Kuhn et al. [22] propose another variational model using again the von Mises yield criterion with a linear hardening term.

Several other phase-field models for ductile fracture have been proposed, which depart in one way or another from the variational framework. Ambati et al. [23] propose a model where the elastic energy is split into a compressive and a tensile part, and the phase-field variable is coupled to plasticity through the elastic degradation function. In [24], the model in [23] is extended to finite strains. Ulloa et al. [25] study the influence of a hardening parameter and the option to consider gradient plasticity with a separate length scale. Miehe et al. [26] consider finite strains in the logarithmic strain space and adapt a history variable to the material properties, including a plasticity threshold on principal tensile stresses. The work by Borden et al. [27] for finite strains comprises an elastic energy splitting, considers von Mises plasticity with a threshold value and shows how stress triaxiality can be introduced through the history variable. In [28], the effect of stress triaxiality is accounted for by incorporating the Gurson-Tvergaard-Needleman (GTN) plasticity model [3]. The phase-field variable evolves based on a history variable that depends on the material porosity and on a given threshold value. In [29], Dittmann et al. propose a phase-field formulation where the effective stress under plastic deformation is determined by the GTN model. In [30], Choo and Sun present a pressure-sensitive fracture model for geomaterials such as porous rock. Drucker-Prager type plasticity is smoothly connected to an elliptical yield surface at high pressure and coupled to the phase-field model through a history variable. Many further phase-field models for ductile fracture have been proposed, including, for example, dynamic loading, the addition of gradient plasticity, various underlying plasticity models, and the degradation of the fracture toughness. Here, we do not aim at covering a full literature review on phase-field modelling for ductile fracture. The review paper [31] by Alessi et al. summarises the underlying theory of phase-field models coupled with plasticity and compares the constitutive equations of existing phase-field models and their numerical results for a uniaxial tension case. The more recent review paper by Marengo and Perego [32] includes theoretical aspects such as the derivation of the strong form equations from the energy functional and from a micromorphic approach.

Summarising, the vast majority of the available models are of non-variational nature. Since their equilibrium and damage evolution equations do not stem from an energy functional, these models enjoy a greater flexibility to reproduce more complex material behaviour, but they give up the theoretical and practical advantages of the variational setting [33]. On the other hand, the few variationally consistent models [18–20, 22], based on von Mises plasticity, are not able to reproduce important phenomenological features of ductile fracture in metals, e.g. triaxiality effects. Even in a specimen loaded in uniaxial tension, when necking starts to take place during plastic yielding a three-dimensional stress state occurs, in which hydrostatic stresses are superposed to axial stresses [6]. The more pronounced the neck, the larger the hydrostatic stresses, which increases the resulting stress level in the stress–strain response [6, 34]. This highlights the importance of considering triaxiality effects for the accurate prediction of ductile fracture when necking occurs. These effects are even more pronounced for complex geometries with notches, where additional hydrostatic stresses already occur in the elastic stage of the loading process.

In this paper, we propose a variational phase-field model coupled with plasticity that can capture with some flexibility stress triaxiality effects relevant to ductile fracture initiation. Our plasticity formulation is based on the modified Cam-Clay plasticity model, that dates back to Roscoe and Burland [35] and is widely used in geomechanics, where it is applied to describe the compressive behaviour of fine-grained soils such as sand, silt and clay [36–40], but may also be used to describe failure of other materials, e.g. snow [41]. As we show in this paper, this plasticity model may also be relevant for other materials such as metals, where the stress triaxiality plays a role in ductile fracture behaviour due to void evolution during plastic deformation. Unlike in standard von Mises plasticity, the yield function of the modified Cam-Clay plasticity model depends on the deviatoric and the hydrostatic stresses, whereby the influence of the hydrostatic stresses can be adjusted with a parameter. Hence, we describe stress triaxiality dependent ductile fracture by coupling modified Cam-Clay plasticity with a phase-field model for fracture. The proposed variational model is restricted to geometrically linear deformations. Varying the intensity of the influence of stress triaxiality is limited to adjusting a single parameter mentioned above, which is included in the formulation through the yield surface.

The paper is structured as follows. In Sect. 2 we first recall the modified Cam-Clay plasticity model, and then formulate the proposed phase-field model coupled with plasticity and its numerical implementation in the commercial FE program Abaqus/Standard  (2022). Analytical homogeneous material responses are presented in Sect. 3. Section 4 illustrates and discusses the numerical results for two-dimensional plane-strain geometries and axisymmetric tensile specimens. Conclusions are drawn in Sect. 5.

In terms of notation, we denote by $\dot{(\cdot )}=d(\cdot )/dt$ the derivative of a quantity with respect to time *t* and by $\partial _{\varvec{x}}{(\cdot )}=\partial (\cdot )/\partial \varvec{x}$ the partial derivative with respect to any other variable $\varvec{x}$. Also, we denote with $\cdot$ the inner product between same-order tensors, as $\Vert \varvec{\sigma }\Vert ^2=\varvec{\sigma }\cdot \varvec{\sigma }$ the (squared) Frobenius norm of the second-order tensor $\varvec{\sigma }$, and as $\varvec{I}_3\in \mathbb R^{3,3}$ the identity matrix. For better clarity, we use round brackets for functional dependencies and square brackets for mathematical operations.

## Formulation of the phase-field model coupled with plasticity

Let us consider a body occupying a domain $\Omega \subseteq \mathbb R^3$ and denote by $\varvec{u}:[0,T]\times \Omega \rightarrow \mathbb {R}^3\,$ its time-dependent displacement field. We assume the body to be mechanically loaded in quasi-static conditions by a hard device imposing a displacement $\varvec{u}=\bar{\varvec{u}}(t)$ on a part of the boundary, $\partial \Omega _{\textsc {d}}$. We adopt a geometrically linear deformation theory, denoting by ${\varvec{\varepsilon }}(\varvec{u}):=\nabla _{sym}\,\varvec{u}$ the linearised strain tensor, which is additively decomposed into

1 $$\begin{aligned} {\varvec{\varepsilon }}={\varvec{\varepsilon }}^e+{\varvec{\varepsilon }}^p\,, \end{aligned}$$

where ${\varvec{\epsilon }}^e$ denotes the elastic and ${\varvec{\epsilon }}^p$ the plastic contribution. The elastic strain energy density and the strain–stress relationships are

2 $$\begin{aligned}& \psi _e({\varvec{\varepsilon }}-{\varvec{\varepsilon }}^p)=\frac{1}{2}\,\mathbb {C}\,[{\varvec{\varepsilon }}-{\varvec{\varepsilon }}^p]\cdot [{\varvec{\varepsilon }}-{\varvec{\varepsilon }}^p]\,,\\& \varvec{\sigma }={\partial _{{\varvec{\varepsilon }}^e} \psi _e({\varvec{\varepsilon }}^e)} = \mathbb {C}\,{\varvec{\varepsilon }}^e \end{aligned}$$

with $\mathbb {C}$ as the fourth-order elasticity tensor.

In the following, we use the decomposition of the stress tensor $\varvec{\sigma }$ in the volumetric part, $\varvec{\sigma }_{vol}=\sigma _{\textsc {h}}\,\varvec{I}_3$, with $\sigma _{\textsc {h}}:=\,\text{tr}\,\varvec{\sigma }/3$ as the hydrostatic stress, and the deviatoric part, $\varvec{\sigma }_{dev}=\varvec{\sigma }-\varvec{\sigma }_{vol}$.

### Modified Cam-Clay plasticity criterion with linear hardening

We consider a modified Cam-Clay plasticity criterion where the admissible stresses $\varvec{\sigma }$ are contained in a convex domain $\mathcal {K}(p):=\{\varvec{\sigma }\,\vert \,f_y(\varvec{\sigma },p)\le 0\}$ defined by a yield function [40, 43–45]

3 $$\begin{aligned} f_y(\varvec{\sigma },p)=\sigma _{eq,\textsc {cc}}(\varvec{\sigma })-\sigma _y(p), \end{aligned}$$

where *p* is the cumulated plastic strain (to be defined later),

4 $$\begin{aligned}&\sigma _{eq,\textsc {cc}}(\varvec{\sigma }): =\sqrt{\frac{3}{2}\,\Vert \varvec{\sigma }_{dev}\Vert ^2+M^2\,\sigma _{\textsc {h}}^2}, \\ &\sigma _y(p)= \sigma _0+H\,p \end{aligned}$$

are respectively the equivalent stress and the yield stress considering linear isotropic hardening; $M>0$ is the hydrostatic parameter, $\sigma _0$ is the initial yield stress and $H\ge 0$ is the hardening modulus. Thus, the yield function for modified Cam-Clay plasticity depends on the von Mises equivalent stress $\sigma _{eq,\textsc {vm}}=\sqrt{{3}/{2}}\,\Vert \varvec{\sigma }_{dev}\Vert$ and on the hydrostatic stress $\sigma _\textsc {h}$; hence, it takes into account the stress triaxiality characterised by $\eta ^*=\sigma _\textsc {h}/\sigma _{eq,\textsc {vm}}$.

**Fig. 1:**
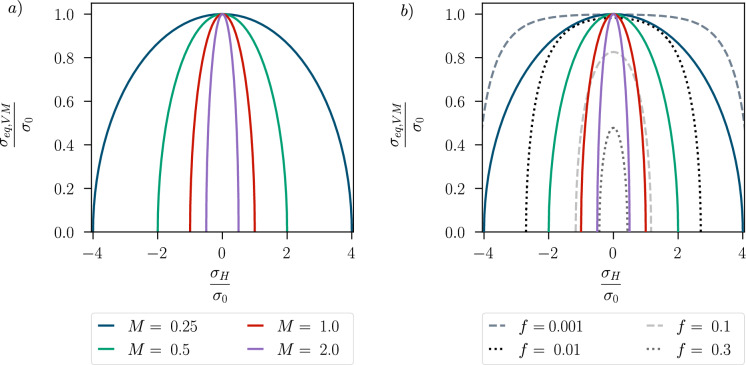
**a** Modified Cam-Clay initial yield surface for different values of *M*, plotted in the plane of the equivalent stress $\sigma _{eq,\textsc {vm}}$ and the hydrostatic stress $\sigma _\textsc {h}$, normalised by the initial yield stress $\sigma _0$; **b** Comparison of the modified Cam-Clay and GTN initial yield surfaces for different porosity values *f* with the GTN parameters $q_1=1.74$, $q_2=1.0$, $q_3=q_1^2$. The colour code of the yield surfaces for the modified Cam-Clay model corresponds to the legend in a)

From (3) and (4), it is evident that the function $f_y$ describes an elliptic yield surface in the $\sigma _{\textsc {h}}$-$\Vert \varvec{\sigma }_{dev}\Vert$-plane with centre in the origin, see Fig. 1a). Unlike the standard von Mises yield surface, which describes a cylinder along the hydrostatic axis in the three-dimensional principal stress space and where the yielding is independent of the hydrostatic stresses, in the modified Cam-Clay plasticity model yielding can take place in a purely hydrostatic stress state. Modified Cam-Clay plasticity thus shows some ability to account for different stress states that arise with differently shaped geometries and different loading scenarios. The yield surface shows some similarity with the one of the well-known GTN plasticity model [2, 3, 46–48], see Fig. 1b). This work considers the same behaviour in compression and tension, as is the case for the GTN model. The radii of the ellipse on the normalised $\sigma _{eq,\textsc {vm}}$ and $\sigma _\textsc {h}$ axes are equal to 1 and 1/*M*, respectively; hence, the hydrostatic parameter $M>0$, specifying the degree of influence of the hydrostatic stress, affects the shape of the yield surface as shown in Fig. 1a). In the limit $M\rightarrow 0$, one recovers the classical von Mises plasticity model. In Fig. 1b), the yield surface for different values of *M* is compared to the yield surface of the GTN model that shrinks for a growing porosity *f*.

### Incremental variational formulation of the modified Cam-Clay plasticity model with linear hardening

We consider an associated plasticity model. Following the framework of generalised standard materials [49], we introduce the plastic dissipation potential $\pi _{\mathcal {K}(p)}(\dot{{\varvec{\varepsilon }}}^p)$ and the convex set $\mathcal {K}(p):=\lbrace \varvec{\sigma }\,\vert \,f_y(\varvec{\sigma },p)\le 0\rbrace$ of admissible stresses [50]. Considering the yield function in (3), for the modified Cam-Clay model the rate of plastic energy density is given by

5 $$\begin{aligned} \pi _{\mathcal {K}(p)}(\dot{{\varvec{\varepsilon }}}^p):=\sup _{\varvec{\sigma }\in \mathcal {K}(p)}\varvec{\sigma }\cdot \dot{{\varvec{\varepsilon }}}^p\,. \end{aligned}$$

Introducing the following Cam-Clay *M*-dependent norm for the stress and the corresponding dual norm for the plastic strain rate as

6 $$\begin{aligned} \Vert \varvec{\sigma }\Vert _{\textsc {m}}&:=\sigma _{eq,\textsc {cc}}(\varvec{\sigma })=\sqrt{{\frac{3}{2}\,\Vert \varvec{\sigma }_{dev}\Vert ^2+M^2\,\sigma _{\textsc {h}}^2}}\,,\\ \Vert \dot{{\varvec{\varepsilon }}}^p\Vert _{\textsc {m}}^*&:=\sup _{\Vert \varvec{\sigma }\Vert _{\textsc {m}}\le \sigma _y(p)}\frac{\varvec{\sigma }}{\sigma _y(p)}\cdot \dot{{\varvec{\varepsilon }}}^p=\sup _{\Vert \tilde{\varvec{\sigma }}\Vert _{\textsc {m}}\le 1}\tilde{\varvec{\sigma }}\cdot \dot{{\varvec{\varepsilon }}}^p \end{aligned}$$

with $\tilde{\varvec{\sigma }}:=\varvec{\sigma }/\sigma _y(p)$, the rate of plastic energy density can be rewritten as follows

7 $$\begin{aligned} \pi _{\mathcal {K}(p)}(\dot{{\varvec{\varepsilon }}}^p)&:=\sup _{\Vert \varvec{\sigma }\Vert _{\textsc {m}}\le \sigma _y(p)}\varvec{\sigma }\cdot \dot{{\varvec{\varepsilon }}}^p\\&\,=\sigma _y(p)\sup _{\Vert \tilde{\varvec{\sigma }}\Vert _{\textsc {m}}\le 1}\tilde{\varvec{\sigma }}\cdot \dot{{\varvec{\varepsilon }}}^p\\&\,= (\sigma _0+H p)\,\dot{p}\,, \end{aligned}$$

where

8 $$\begin{aligned} \dot{p} =\Vert \dot{\varvec{\varepsilon }}^p\Vert _{\textsc {m}}^* =\sqrt{ \frac{2}{3}\,\Vert \dot{{\varvec{\varepsilon }}}^p_{dev}\Vert ^2+\frac{\text{tr}^2\,\dot{{\varvec{\varepsilon }}}^p}{M^2}} \end{aligned}$$

is the cumulated plastic strain rate. The explicit computation of the dual norm requires the solution of the minimisation problem in the definition of the dual norm, see Appendix A. The potential $\pi _{\mathcal {K}(p)}$ in (7) is composed of the dissipation potential and the hardening term, considered as the rate of plastic free energy density. Another approach would be to derive $\pi _{\mathcal {K}(p)}$ as dissipation potential only, subtracting the plastic part of the Helmholtz free energy from the rate of plastic work [51]. In this case, $\pi _{\mathcal {K}(p)}$ is additionally a function of $\dot{p}$, where *p* is introduced as an additional state variable, see [50], and the hardening term is added to the free elastic energy density. Both derivation approaches yield the same plastic energy given below, which is the relevant term for the proposed model.

The plastic energy density is the integral in time of the rate of plastic energy density. It is a function of the cumulated plastic strain *p* only

9 $$\begin{aligned} \psi _{p}(p):=\int _0^t\pi _{\mathcal {K}(p)}(\dot{{\varvec{\varepsilon }}}^p(\tau ))\,\text{d}\tau =\sigma _0\,p+\frac{1}{2}\,H\,p^2\,,\quad p=\int _0^t\Vert \dot{\varvec{\varepsilon }}^p(\tau )\Vert _{\textsc {m}}^*\,\text{d}\tau \,. \end{aligned}$$

Considering a body $\Omega$ under quasi-static loading in the form of imposed displacements on the Dirichlet part of the boundary $\partial \Omega _{\textsc {d}}$, the total energy of the body obeying the modified Cam-Clay plasticity model is defined as the sum of the elastic energy and the plastic energy

10 $$\begin{aligned} \mathcal {E}(\varvec{u},{\varvec{\varepsilon }}^p,p)=\int _\Omega \psi _e({\varvec{\epsilon }}(\varvec{u})-{\varvec{\varepsilon }}^p)\,\text{d}\varvec{x}+\int _\Omega \psi _p(p)\,\text{d}\varvec{x}\,, \end{aligned}$$

which is a function of the state variables $\varvec{u}$, ${\varvec{\varepsilon }}^p$, and *p*.

In the time-discrete setting, given the solution $(\varvec{u}_{i-1},{\varvec{\varepsilon }}^p_{i-1},p_{i-1})$ at the time step $t_{i-1}$, the cumulated plastic strain at the next time step $t_i=t_{i-1}+\Delta t$ can be expressed using a backward Euler scheme as

11 $$\begin{aligned} p_i=p_{i-1}+\Vert \Delta {\varvec{\varepsilon }}^p_i\Vert ^*_{\textsc {m}}\,, \end{aligned}$$

where $\Delta {\varvec{\varepsilon }}^p_i:={\varvec{\varepsilon }}^p_i-{\varvec{\varepsilon }}^p_{i-1}$ is the increment of the plastic strain between the two steps. The solution of the time-discrete elasto-plastic evolution problem can be obtained by solving the following incremental variational problem for the displacement $\varvec{u}_i$ and the plastic strain ${\varvec{\varepsilon }}^p_i$ at the *i*-th time step

12 $$\begin{aligned} (\varvec{u}_i,{\varvec{\varepsilon }}^p_i)\in \mathop {\text {argmin}}\limits _{\varvec{u}\in \mathcal {V}_i,{\varvec{\varepsilon }}^p\in \mathcal {P}} \mathcal {E}_i(\varvec{u},{\varvec{\varepsilon }}^p)\,, \end{aligned}$$

where the total energy at time $t_{i}$ is given by

13 $$\begin{aligned} \mathcal {E}_i(\varvec{u},{\varvec{\varepsilon }}^p):= \int _\Omega \psi _e({\varvec{\varepsilon }}(\varvec{u})-{\varvec{\varepsilon }}^p)\,\text{d}\varvec{x}+\int _\Omega \psi _p\left( p_{i-1}+\Vert {\varvec{\varepsilon }}^p-{\varvec{\varepsilon }}^p_{i-1}\Vert _{\textsc {m}}^*\,\right) \,\text{d}\varvec{x}\,, \end{aligned}$$

and, assuming work hardening, the spaces of the admissible displacements and plastic strain tensors are

14 $$\begin{aligned} \mathcal {V}_i :=\{\tilde{\varvec{u}}\in H^1(\Omega ,\mathbb {R}^3)\,\vert \, \tilde{\varvec{u}}=\bar{\varvec{u}}_i\,\text {on}\,\partial _\textsc {d}\Omega \},\quad \mathcal {P}:=\{\tilde{{\varvec{\varepsilon }}}^p\in L^2(\Omega ,\mathbb {R}^{3,3})\}, \end{aligned}$$

where $L^2$ and $H^1$ denote respectively the spaces of square integrable functions and square integrable functions with square integrable weak derivatives [15–17, 52]. From now on, for notational simplicity, we omit the subscript *i* for all quantities related to the current time step $t=t_i$.

The time-discrete elasto-plastic evolution equations at time $t_{i}$ are the first-order optimality conditions of the minimisation problem (12), see Appendix B.

- The stationarity conditions with respect to the displacement field $\varvec{u}$ yield the strong form equilibrium equations and boundary conditions for the Cauchy stress $\varvec{\sigma }$
  15 $$\begin{aligned} \text{div}\,{\varvec{\sigma }}=\textbf{0}\quad \text {in }\Omega ,\quad \varvec{\sigma }\,\varvec{\nu }=\textbf{0}\quad \text {on }\,\partial \Omega _{\textsc {n}}\,, \end{aligned}$$
  where the boundary decomposes as $\partial \Omega =\partial \Omega _{\textsc {d}}\cup \partial \Omega _{\textsc {n}}$ and $\varvec{\nu }\in \mathbb {R}^3$ denotes the unit outer normal vector.
- The stationarity conditions with respect to the plastic strain ${\varvec{\varepsilon }}^p$ imply (see Appendix B)
  16 $$\begin{aligned} \Delta {\varvec{\varepsilon }}^p=\Delta \lambda \,\frac{\partial f_y}{\partial {\varvec{\sigma }}},\quad \Delta \lambda \ge 0,\\ f_y(\varvec{\sigma },p)\le 0,\quad f_y(\varvec{\sigma },p)\,\Delta \lambda =0, \end{aligned}$$
  with $\Delta \lambda =\Vert \Delta {\varvec{\varepsilon }}^p\Vert ^*_{\textsc {m}}$, i.e. the classical normality rule and Karush-Kuhn-Tucker conditions of elasto-plasticity. Moreover, defining the elastic predictor stress as
  $$\varvec{\sigma }^{tr}:=\varvec{\sigma }_{i-1}+\mathbb {C}\,[{\varvec{\varepsilon }}-{\varvec{\varepsilon }}_{i-1}] = K\,\text{tr}({\varvec{\varepsilon }}-{\varvec{\varepsilon }}^{p}_{i-1})\varvec{I}_3 + 2\mu [{\varvec{\varepsilon }}_{dev}-{\varvec{\varepsilon }}_{dev,i-1}^{p}],$$
  where $\mu =E/[2\,[1+\nu ]]$ denotes the shear modulus, $K=E\,\nu /[[1+\nu ]\,[1-2\,\nu ]]+2/3\,\mu$ the bulk modulus and $\nu$ the Poisson’s ratio, yields
  - if $$\Vert \varvec{\sigma }^{tr}\Vert _{\textsc {m}}\le \sigma _y(p_{i-1})$$, $$\Delta {\varvec{\varepsilon }}^p=\textbf{0}$$;
  - if $\Vert \varvec{\sigma }^{tr}\Vert _{\textsc {m}}>\sigma _y(p_{i-1})$, the incremental relations (16) can be integrated by solving the following nonlinear scalar equations for $\text{tr}(\Delta {{\varvec{\varepsilon }}}^p)$ and $\Vert \Delta {{\varvec{\varepsilon }}}^p_{dev}\Vert$
    17 $$\begin{aligned} \left[ \frac{1}{M^2}\left[ \frac{\,\sigma _y(p_{i-1})}{\,\Vert \Delta {\varvec{\varepsilon }}^p\Vert _{\textsc {m}}^*}+H\right] +K \right] \text{tr}(\Delta {{\varvec{\varepsilon }}}^p)= & \frac{1}{3}\text{tr}(\varvec{\sigma }^{tr}) \end{aligned}$$
    18 $$\begin{aligned} 2\left[ \frac{1}{3}\left[ \frac{\,\sigma _y(p_{i-1})}{\,\Vert \Delta {\varvec{\varepsilon }}^p\Vert _{\textsc {m}}^*}+H\right] +\,\mu \,\right] \Vert \Delta {{\varvec{\varepsilon }}}^p_{dev}\Vert= & \Vert \varvec{\sigma }^{tr}_{dev}\Vert . \end{aligned}$$
    The tensors $\varvec{\sigma }$, $\Delta {\varvec{\varepsilon }}^p$, and $\varvec{\sigma }^{tr}$ are co-axial.
  This return mapping algorithm was already proposed by Aravas [53].

### Phase-field model for ductile fracture based on modified Cam-Clay plasticity

To account for damage in the material, the modified Cam-Clay plasticity model is coupled with a phase-field fracture model. The phase-field variable representing the damage in the material is denoted by $d:[0,T]\times \Omega \longrightarrow [0,1]$, whereby $d=0$ and $d=1$ respectively describe the intact and the completely damaged material states. The incremental variational functional of the coupled model extends the formulation of the plasticity model in the previous section by adding the damage contribution, following [18]. The total energy of the body at time $t_i$ reads as

19 $$\begin{aligned} \mathcal {E}_i(\varvec{u},{\varvec{\varepsilon }}^p,d)=&\int _\Omega g(d)\,\psi _e({\varvec{\varepsilon }}(\varvec{u})-{\varvec{\varepsilon }}^p)\,\text{d}\varvec{x}\,\nonumber \\  &+\int _\Omega \rho (d)\,\psi _p\left( p_{i-1}+\Vert {\varvec{\varepsilon }}^p-{\varvec{\varepsilon }}^p_{i-1}\Vert _{\textsc {m}}\,\right) \,\text{d}\varvec{x}\, \\&+ \int _\Omega \frac{G_c}{c_w}\left[ \frac{w(d)}{\ell }+ \ell \,\Vert \nabla d\Vert ^2\right] \,\text{d}\varvec{x}\,, \end{aligned}$$

where the elastic and plastic energies are coupled to the damage via the degradation functions *g* and $\rho$, respectively, and the last integral represents the damage energy. In this work, we consider standard quadratic degradation functions [13, 32], i.e. $g,\rho :[0,1]\longrightarrow \mathbb R$ given by

20 $$\begin{aligned} g(d)=(1-d)^2\,,\quad \rho (d)=(1-d)^2\,. \end{aligned}$$

The parameter $\ell >0$ denotes the internal length that controls the width of the damage zone, $G_c$ represents the fracture toughness and $c_w$ is a scaling parameter given by [54]

21 $$\begin{aligned} c_w:=4\,\int _0^1 w(s)^\frac{1}{2}\,\text{d}s\,. \end{aligned}$$

In the following, we only consider the AT1-model [31, 55] as it allows for an initial purely elastic and plastic stage without damage evolution. Hence, the dissipation function is defined by

22 $$\begin{aligned} w:[0,1]\longrightarrow \mathbb R_+\,,\quad w(d)=\,d\,, \end{aligned}$$

resulting in $$c_w=8/3$$.

To ensure the irreversibility of the damage process, the condition $d_i(\varvec{x})\ge d_{i-1}(\varvec{x})$ must apply for all $\varvec{x}\in \Omega$ at all times. We assume *d* is sufficiently regular such that at fixed time $t_i$

$$\begin{aligned}d\in \mathcal {W}_{i}:=\big \lbrace \tilde{d}\in H^1(\Omega )\,\vert \,\tilde{d}\ge d_{i-1} \,,\;0\le \tilde{d}\le 1 \big \rbrace \,.\end{aligned}$$

The solution of the time-discrete elasto-plastic fracture evolution problem can again be obtained by incremental energy minimisation. Given $\varvec{u}_{i-1}$, ${\varvec{\varepsilon }}^p_{i-1}$, $p_{i-1}$ and the phase-field variable $d_{i-1}$ at the previous time step $t_{i-1}$, the solution of the minimisation problem at time $t_i$ is given by

23 $$\begin{aligned} (\varvec{u},{\varvec{\varepsilon }}^p, d)\in \mathop {\text {argmin}}\limits _{\varvec{u}\in \mathcal {V}_i,{\varvec{\varepsilon }}^p\in \mathcal {P},d\in \mathcal {W}_i} \mathcal {E}_i(\varvec{u},{\varvec{\varepsilon }}^p,d)\,. \end{aligned}$$

First-order optimality for the problem above gives the following necessary conditions

- $\varvec{u}$-optimality gives the equilibrium Eq. (15), where the stress is affected by the phase-field variable
  24 $$\begin{aligned} \varvec{\sigma }=g(d){\partial _{{\varvec{\varepsilon }}^e} \psi _e({\varvec{\varepsilon }}^e)} = g(d) \mathbb {C}\,[{\varvec{\varepsilon }}-{\varvec{\varepsilon }}_p]\,. \end{aligned}$$
- ${\varvec{\varepsilon }}^p$-optimality gives the plastic flow rule as in Sect. 2.2 with a damage-dependent yield stress (see [19] and Appendix B)
  - if $$\Vert \varvec{\sigma }^{tr}\Vert _{\textsc {m}}\le \sigma _y(p_{i-1},d)$$, $$\Delta {\varvec{\varepsilon }}^p=\textbf{0}$$;
  - if $\Vert \varvec{\sigma }^{tr}\Vert _{\textsc {m}}>\sigma _y(p_{i-1},d)$, $\Delta {\varvec{\varepsilon }}^p$ must verify
    25 $$\begin{aligned} \left[ \frac{\rho (d)}{M^2}\left[ \frac{\,\sigma _y(p_{i-1})}{\,\Vert \Delta {\varvec{\varepsilon }}^p\Vert _{\textsc {m}}^*}+H\right] +g(d)\,K \right] \text{tr}(\Delta {{\varvec{\varepsilon }}}^p)= & \frac{1}{3}\text{tr}(\varvec{\sigma }^{tr}) \end{aligned}$$
    26 $$\begin{aligned} 2\left[ \frac{\rho (d)}{3}\left[ \frac{\,\sigma _y(p_{i-1})}{\,\Vert \Delta {\varvec{\varepsilon }}^p\Vert _{\textsc {m}}^*}+H\right] +g(d)\,\,\mu \,\right] \Vert \Delta {{\varvec{\varepsilon }}}^p_{dev}\Vert= & \Vert \varvec{\sigma }^{tr}_{dev}\Vert \end{aligned}$$
    where
    27 $$\begin{aligned} \sigma _y(p,d)&:= \rho (d)\psi _p'(p)=\rho (d)\sigma _y(p)\\&\,= \rho (d)[\sigma _y(p_{i-1})+H \Vert \Delta {\varvec{\varepsilon }}^p\Vert _{\textsc {m}}^*]. \end{aligned}$$
    The optimality conditions with respect to ${\varvec{\epsilon }}^p$ can be summarised as Karush-Kuhn-Tucker conditions as in (16) with the damage-dependent yield function given by
    28 $$\begin{aligned} f_y(\varvec{\sigma },p,d)&=\sigma _{eq,\textsc {cc}}(\varvec{\sigma })-\sigma _y(p,d)\\&=\Vert \varvec{\sigma }\Vert _{\textsc {m}}-\sigma _y(p,d)\,. \end{aligned}$$
- *d*-optimality, under the irreversibility condition, gives the phase-field evolution equations (see [54]) in the form of Karush-Kuhn-Tucker complementarity conditions
  29 $$\begin{aligned} \begin{aligned} f_{d}(\varvec{\sigma },p,d)\le 0\,,\quad d-d_{i-1}\ge 0\,,\quad f_{d}(\varvec{\sigma },p,d)\,[d-d_{i-1}]=0\qquad \text {in }\Omega \,,\\ \nabla d\cdot \varvec{\nu }\ge 0\,,\quad \nabla d\cdot \varvec{\nu }\,[d-d_{i-1}]=0\qquad \text {on }\partial \Omega \,, \end{aligned} \end{aligned}$$
  where
  30 $$\begin{aligned} \begin{aligned} \qquad f_{d}(\varvec{\sigma },p,d):=s^\prime ( d )\,\ \psi ^*_e(\varvec{\sigma }) - \rho ^\prime ( d )\,\psi _{p}(p) - \frac{G_c}{c_w}\,\left[ \frac{1}{\ell }-2\,\ell \,\bigtriangleup d \right] \, \end{aligned}\end{aligned}$$
  with $s(d):=1/g(d)$ and the complementary elastic energy density
  31 $$\begin{aligned} \psi ^*_e(\varvec{\sigma }):=\frac{1}{2}\,\mathbb {C}^{-1}\,\varvec{\sigma }\cdot \varvec{\sigma }. \end{aligned}$$

### Numerical implementation of the phase-field model coupled with modified Cam-Clay plasticity

The numerical implementation is performed in Abaqus/Standard (2022 version) by a time-implicit coupled temperature-displacement analysis [42]. To implement the proposed material model, as suggested in  [56, 57], we deploy the user subroutines Umat and Hetval, which contain the plasticity return map and the strong form of the evolution equation for the phase-field variable, respectively. The built-in mechanical solver of Abaqus calculates the displacement and strain increments, and the phase-field increments are determined by the thermal solver using the analogy to the heat equation. To keep the formulation variationally consistent, as proposed in [58], the irreversibility of the phase-field variable at time $t_{i}$ is enforced approximately by adding the penalty term

32 $$\begin{aligned} \frac{\gamma }{2}\,\int _\Omega \,\langle d-d_{i-1}\rangle _-^2\,\text{d}\varvec{x} \end{aligned}$$

to the energy functional $\mathcal {E}_{i}$ in (19), where $\langle \cdot \rangle _-:=\min (\cdot ,\,0)$. At time step $t_i$, the minimisers of the unconstrained optimisation problem (23), which includes the additional penalty term in (32), are found by applying a staggered solution scheme, see Box 1. To compute the displacement and the phase-field variable at $t_{i}$, several iterations *k* are performed.

At time $t_{i}$ and iteration *k*, the Umat subroutine computes the trial stress $\varvec{\sigma }^{tr,k}=\varvec{\sigma }_{{i-1}}+g(d^{k-1})\,\mathbb {C}\,\Delta {\varvec{\varepsilon }}^k$ and the yield criterion is checked by computing the yield function in (28), $f_y(\varvec{\sigma }^{tr,k}, p_{i-1}, d^{k-1})=\Vert \varvec{\sigma }^{tr,k}\Vert _{\textsc {m}}-\sigma _y(p_{i-1},d^{k-1})$. If the yield criterion is violated, the return map algorithm [53] numerically solves (25), (26) for $\text{tr}(\Delta {\varvec{\varepsilon }}^{p,k})$, $\Vert \Delta {\varvec{\varepsilon }}_{dev}^{p,k}\Vert$. To this end, the Newton-Raphson method is applied to iteratively solve the equivalent system of equations

33 $$\begin{aligned} \begin{pmatrix}\frac{3}{2}\,\Vert \varvec{\sigma }_{dev}^k\Vert \,\text{tr}(\Delta {\varvec{\varepsilon }}^{p,k})-M^2\,\frac{\text{tr}(\varvec{\sigma }^k)}{3}\,\Vert \Delta {\varvec{\varepsilon }}_{dev}^{p,k}\Vert \\ f_y(\varvec{\sigma }^k,p^k,d^k)\end{pmatrix}=\textbf{0}\end{aligned}$$

for the plastic strain increments, where the first equation results from combining (25) and (26). As function of $\text{tr}(\Delta {\varvec{\varepsilon }}^{p,k})$ and $\Vert \Delta {\varvec{\varepsilon }}_{dev}^{p,k}\Vert$, the plastic strain tensor at iteration *k* is given by

34 $$\begin{aligned} {\varvec{\varepsilon }}^{p,k}= {\varvec{\varepsilon }}_{i-1}^p+\frac{1}{3}\,\text{tr}(\Delta {\varvec{\varepsilon }}^{p,k})\,\varvec{I}_3+\Vert \Delta {\varvec{\varepsilon }}_{dev}^{p,k} \Vert \,\textbf{n}^k\,, \end{aligned}$$

where we define the director $\textbf{n}^k:=\Delta {\varvec{\varepsilon }}_{dev}^{p,k} /\Vert \Delta {\varvec{\varepsilon }}_{dev}^{p,k} \Vert$. According to (11), the cumulated plastic strain is given by

35 $$\begin{aligned} p^k= p_{i-1}+\Vert \Delta {\varvec{\epsilon }}^{p,k}\Vert ^*_{\text{M}}\, \end{aligned}$$

and the stress tensor by

36 $$\begin{aligned} \varvec{\sigma }^k=\varvec{\sigma }^{tr,k}-g(d^{k-1})\,K \,\text{tr}(\Delta {\varvec{\varepsilon }}^{p,k})\,\varvec{I}_3-g(d^{k-1})\,2\,\mu \,\Vert \Delta {\varvec{\varepsilon }}_{dev}^{p,k}\Vert \,\textbf{n}^k.\, \end{aligned}$$

After solving (33), the consistent tangent operator $\mathbb {C}^{t,k}$ is updated and passed to the internal solver.

The increment of the phase-field variable $\Delta d^k$ at iteration *k* is determined by exploiting the analogy of the strong form evolution equation for the phase-field $f_d(\varvec{\sigma },p,d)=0$, see (30), to the heat equation [56, 57]. We first determine the complementary elastic energy density in (31) and the plastic energy density in (9) as follows

37 $$\begin{aligned} \psi _{e}^{*, k}=\frac{\varvec{\sigma }_{dev}^k\cdot \varvec{\sigma }_{dev}^k}{4\,\mu }+\frac{(\sigma _{\textsc {h}}^k)^2}{2\,K},\quad \psi _{p}^k=\sigma _0\, p^k+\frac{1}{2}\,H\,( p^k)^2\, \end{aligned}$$

and then use them to compute the input required by the subroutine Hetval, i.e. the flux

38 $$\begin{aligned} q\! :=& -\ell ^2 \bigtriangleup d^{k-1} \\ = & -\frac{\ell }{2}\,\frac{c_w}{G_c}\,\left[ -s^\prime (d^{k-1})\,\psi _{e}^{*, k}+\rho ^\prime (d^{k-1})\,\psi _{p}^k\right] \\ & -\frac{1}{2}-S_{opt}\,\langle d^{k-1}-d_{i-1}\rangle _-\,, \end{aligned}$$

corresponding to the discrete form of $f_d(\varvec{\sigma },p,d)=0$, with the constant [58]

39 $$\begin{aligned} S_{opt}=\frac{\ell }{2}\,\frac{c_w}{G_c}\,\gamma =\frac{9}{16\,\textsc {Tol}_{ir}}\,. \end{aligned}$$

The tolerance threshold for the irreversibility of the phase-field variable is set to $\textsc {Tol}_{ir}=0.001$ in order to obtain a sufficiently large penalty parameter. The thermal solver in Abaqus uses the flux in (38) to determine $\Delta d^k$.

**Box 1:**
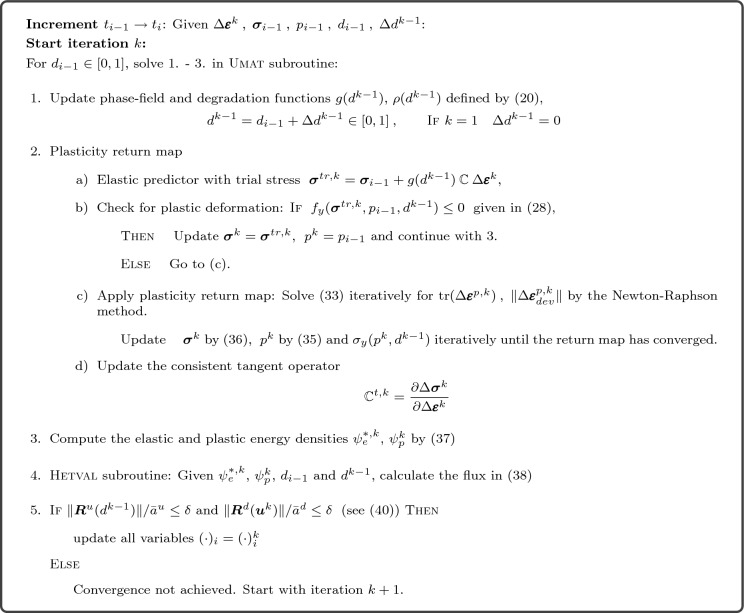
Numerical implementation of modified Cam-Clay plasticity coupled with the phase-field model in Abaqus/Standard (2022) using a staggered approach. The tolerance for convergence of the Newton–Raphson method in the return map is set to $10^{-6}$. The tolerance of the normalised residuals is set to $\delta =0.005$

At every iteration *k*, we solve the equilibrium equations and the phase-field evolution equation as follows

40 $$\begin{aligned} \varvec{K}^{uu}(d^{k-1})\,\Delta \bar{\varvec{u}}^k&=-\varvec{R}^{u}(d^{k-1})\,,\\ \varvec{K}^{dd}(\varvec{u}^{k})\,\Delta \bar{\varvec{d}}^k&=-\varvec{R}^d(\varvec{u}^{k})\,, \end{aligned}$$

where $\varvec{K}^{uu}$, $\varvec{K}^{dd}$ denote the submatrices on the diagonal of the Jacobian matrix of the coupled damage-displacement problem, $\varvec{R}^{u}$ and $\varvec{R}^d$ the corresponding right-hand side (residual) vectors, and $\bar{\varvec{u}}$, $\bar{\varvec{d}}$ the vectors of the nodal displacement and phase-field variable values, respectively. Before proceeding to the next time increment, iterations are performed until the normalised nodal residuals reach a specified tolerance, i.e. $\Vert \varvec{R}^u\Vert /\bar{a}^u<\delta _1$ and $\Vert \varvec{R}^d\Vert /\bar{a}^d<\delta _2$. The normalisation constants $\bar{a}^u$ and $\bar{a}^d$ respectively depend on the spatial and time-averaged nodal forces and phase-field fluxes ($-\ell ^2\,\bigtriangleup d_{i}^{k}$). The tolerance is set to $\delta _1=\delta _2=0.005$.

Choosing sufficiently small (pseudo) time steps is crucial for the plasticity return map to converge during the softening stage. In the following simulations, the time step is set to $\Delta t=10^{-4}\text {s}$, corresponding to $10^4$ increments. In addition, a sufficient number of staggered iterations must be allowed for, especially in the plastic and softening stages and at the beginning of the analysis to find the initial phase-field increment. In the following simulations, we need up to about 230 iterations per time increment, depending on the specific input parameters, time step and geometry.

## Homogeneous material responses

In this section, analytical material responses are derived for the proposed model under the assumption of homogeneous damage, starting in one dimension and continuing with the multi-dimensional case.

### One-dimensional analytical solutions

In the one-dimensional case, the yield function reads

41 $$\begin{aligned} f_y(\sigma ,\varepsilon ^p,d )=\sigma -\rho (d)\,[\sigma _0+H\,\varepsilon ^p]\,, \end{aligned}$$

where $\sigma =g(d) \,E\,(\varepsilon -\varepsilon ^p)$ and $E>0$ denotes the Young’s modulus. We assume monotonic loading and thus directly consider the plastic strain $\varepsilon ^p\in \mathbb {R}$ instead of the cumulated plastic strain. The hydrostatic parameter *M* does not play a role in the one-dimensional case, and the model in this particular setting reduces to von Mises plasticity. Due to the condition $f_{y}\,\Delta \lambda =0$ in (16), for evolving plastic strain $\Delta \lambda >0$ it is $f_{y}= 0$. The corresponding value of stress, which we refer to as yield stress $\sigma _y$, is then given by

42 $$\begin{aligned} \sigma _{y}(\varepsilon ^p,d)=\rho (d)\,[\sigma _0+H\,\varepsilon ^p]. \end{aligned}$$

The damage yield function is given by (30) and is expressed for a homogeneous damage state as

43 $$\begin{aligned} \begin{aligned} f_{d}(\sigma ,\varepsilon ^p,d)= \,&s^\prime (d)\,\frac{1}{2}\,E^{-1}\,\sigma ^2 -\rho ^\prime ( d )\,\psi _{p}(\varepsilon ^p)-\frac{G_c}{c_w}\,\frac{1}{\ell }\,.\\ \end{aligned} \end{aligned}$$

Due to the condition $f_{d}\,[d-d_{i-1}]=0$ in (29), for evolving damage $d>d_{i-1}$ it is $f_{d}= 0$. The corresponding value of stress, which we refer to as damage yield stress $\sigma _d$, is then given by

44 $$\begin{aligned} \sigma _d(\varepsilon ^p,d)=\sqrt{E\,[1-d]^3\,\left[ \rho ^\prime ( d )\,\psi _{p}(\varepsilon ^p)+\frac{G_c}{c_w\,\ell }\right] }\,. \end{aligned}$$

For the AT1-model, $\sigma _d$ reaches its maximum value in the elastic stage; this value is given by

45 $$\begin{aligned} \sigma _{d}(0,0)=\sqrt{\frac{3}{8}\,\frac{E\,G_c}{\ell }}\,, \end{aligned}$$

see also the derivations in [59]. Considering again the damage yield function in (43) and the quadratic degradation functions in (20), by solving $f_{d}(\sigma ,\varepsilon ^p,d)=0$ for the phase-field variable we obtain

46 $$\begin{aligned} d(\varepsilon ,\varepsilon ^p)=1-\frac{\frac{G_c}{c_w\,\ell }}{E\,[\varepsilon -\varepsilon ^p]^2+2\,\psi _p(\varepsilon ^p)}\,. \end{aligned}$$

The initiation of damage depends on the constant

47 $$\begin{aligned} \zeta =\frac{1}{\sigma _0}\,\sqrt{\frac{3}{8}\,\frac{E\,G_c}{\ell }}>0\,. \end{aligned}$$

**Fig. 2:**
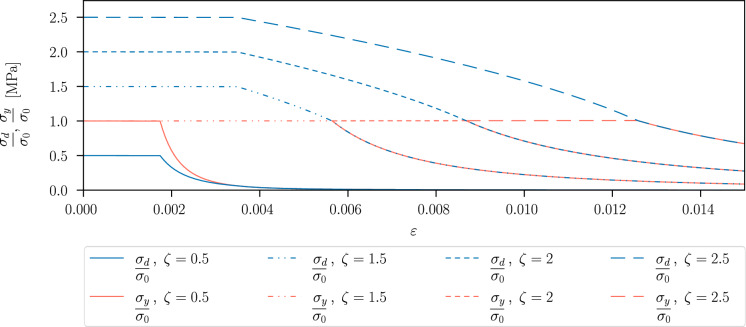
Damage yield stress $\sigma _d$ and plastic yield stress $\sigma _y$, normalised by the initial yield stress $\sigma _0$, as functions of the strain for different values of $\zeta$ given in (47). The material parameters are $E=210~\text {GPa}$, $\sigma _0=730~\text {MPa}$, $H=500~\text {MPa}$, $\ell =0.15~\text {mm}$

For $\zeta <1$, it is $\sigma _d(0,0)<\sigma _y(0,0)$, i.e. the damage yield criterion is reached before the plastic yield criterion during the loading process [20]. Instead, for $\zeta \ge 1$, it is $\sigma _y(0,0)\le \sigma _d(0,0)$, thus the plastic yield criterion is fulfilled first. The behaviour of $\sigma _y$ and $\sigma _d$ is displayed in Fig. 2 for different values of $\zeta$. In the following, we focus on examples where $\zeta \ge 1$, as this reflects the experimentally observed ductile failure behaviour. In this case, a plastic stage is reached, where the plastic strain evolves before the phase-field variable starts to grow. Increasing $\zeta$ leads to a higher material ductility [60]. If all other parameters are kept fixed, a higher value of $\zeta$ is associated with a higher fracture toughness, which increases the initial maximum value of the damage stress $\sigma _{d}(0,0)$ in (45) and hence extends the plastic stage. As a related effect, the higher fracture toughness also leads to a slower phase-field evolution in the softening stage, which becomes visible by a less rapid decrease of the stress in Fig. 2. The slope of the hardening branch appears small because the hardening parameter *H* is relatively small. However, we use this value as it is of the same order of magnitude used for the simulations presented in Sect. 4.

**Fig. 3:**
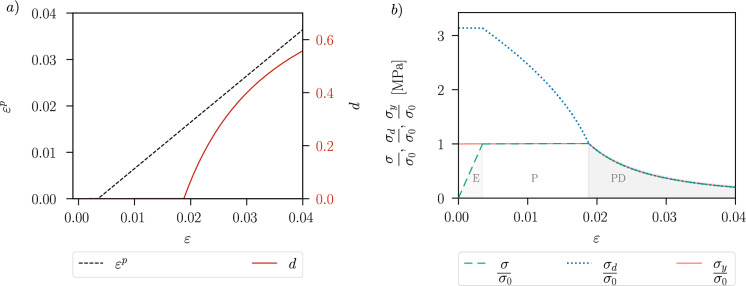
Example of an E-P-PD response, generated with the standard degradation functions $g(d)=(1-d)^2$, $\rho (d)=(1-d)^2$, $E=210~\text {GPa}$, $\sigma _0=730~\text {MPa}$, $H=500~\text {MPa}$, $\ell =0.15~\text {mm}$, $G_c=10~\text {N/mm}$; **a** plastic strain and phase-field evolution with respect to strain, **b** stress–strain response considering the normalised stress, damage yield stress and plastic yield stress

Assuming $\zeta \ge 1$, the homogeneous ductile material response of the proposed model in the one-dimensional case can be divided into the following three stages. These stages are illustrated in Fig. 3, displaying the corresponding evolution of the plastic strain $\varepsilon ^p$, the phase-field variable *d* and the stress–strain curve.

(i) Linear-elastic stage (E), which is guaranteed by the AT1-model, as the damage only starts to evolve after a certain threshold value of the strain/stress. The maximum stress in this stage is given by the initial yield stress $\sigma _0$. The plastic yield stress $\sigma _{y}$ and damage yield stress $\sigma _d$ remain constant during the elastic stage, see Fig. 3b). We have
  $$\begin{aligned}\varepsilon \in \left[ 0,\frac{\sigma _0}{E}\right] \,,\quad \varepsilon ^p\equiv 0\,,\quad d\equiv 0\,,\quad \sigma =E\,\varepsilon \,;\end{aligned}$$
(ii) Plastic stage with isotropic linear hardening (P) in a strain interval of
  $$\begin{aligned}\varepsilon \in \left[ \frac{\sigma _0}{E}\,,\;\left( \frac{1}{E}+\frac{1}{H}\right) \sqrt{\frac{E\,\sigma _0^2+E\,H\,\frac{G_c}{c_w\,\ell }}{H+E}}-\frac{\sigma _0}{H} \right] \,.\end{aligned}$$
  The plastic yield criterion $f_y(\sigma ,\varepsilon ^p,d)=0$ is fulfilled in this stage, and plastic strain starts to evolve. Due to the plastic strain evolution, $\sigma _d$ decreases during the plastic stage, while the damage yield criterion is still not met since we consider the case $\zeta \ge 1$. The plastic strain, the phase-field variable and the stress are given by
  $$\begin{aligned} \varepsilon ^p=\frac{E\,\varepsilon -\sigma _0}{E+H}\,, \quad d\equiv 0\,,\quad \sigma =\sigma _{y}(\varepsilon ^p,d)\,;\end{aligned}$$
(iii) When the plastic yield stress $\sigma _{y}$ and the damage yield stress $\sigma _{d}$ intersect at a plastic strain $\hat{\varepsilon } ^p>0$, i.e.
  48 $$\begin{aligned} \sigma _{y}(\hat{\varepsilon }^p,0)=\sigma _d( \hat{\varepsilon }^p,0)\,, \end{aligned}$$
  the phase-field variable *d* starts to evolve and leads to material softening [20]. This stage occurs in a strain interval of
  $$\begin{aligned}\varepsilon \in \left[ \left( \frac{1}{E}+\frac{1}{H}\right) \sqrt{\frac{E\,\sigma _0^2+E\,H\,\frac{G_c}{c_w\,\ell }}{H+E}}-\frac{\sigma _0}{H}\,,\;\infty \right] \,.\end{aligned}$$
  The minimum of the strain interval is determined by $\varepsilon =\varepsilon ^e+\varepsilon ^p$ with the elastic strain given by $\varepsilon ^e=\sigma _{y}(\varepsilon ^p,0)/E$ and the plastic strain given by the condition in (48). A distinction is made between damage with constant plastic strain (D) and coupled plasticity and damage (PD). With regard to practical applications with pronounced ductile material behaviour, in the examples of this work we focus on the (E-P-PD) response, where damage and plasticity both evolve after an elastic and a purely plastic stage. The choice of identical degradation functions $g(d)=\rho (d)$ precludes the (E-P-D) response. In the case of the plastic-damage stage (PD), plasticity continues to evolve when the phase-field *d* starts to evolve [20], since the yield criteria $f_{y}(\sigma ,\varepsilon ^p,d)=0$ and $f_{d}(\sigma ,\varepsilon ^p,d)=0$ both apply. We have
  $$\begin{aligned}\varepsilon ^p=\frac{E\,\varepsilon -\sigma _0}{E+H}\,,  d(\varepsilon ,\varepsilon ^p)=1-\frac{\frac{G_c}{c_w\,\ell }}{\frac{\sigma _{y}(\varepsilon ^p,0)^2}{E}+2\,\psi _p(\varepsilon ^p)}\,, \sigma =\sigma _{y}(\varepsilon ^p,d)\,.\end{aligned}$$
  As shown in Fig. 3b), for the given parameters, the condition in (48) is fulfilled with $\hat{\varepsilon }^p=0.015$ at a total strain of $\varepsilon =0.018$. That is, the damage yield stress $\sigma _d$ meets the plastic yield stress $\sigma _{y}$ at $\sigma /\sigma _0=1.01$, and this marks the beginning of softening, where $\sigma _d(\varepsilon ^p,d)=\sigma _{y}(\varepsilon ^p,d)$.

### Multi-dimensional material behaviour

**Fig. 4:**
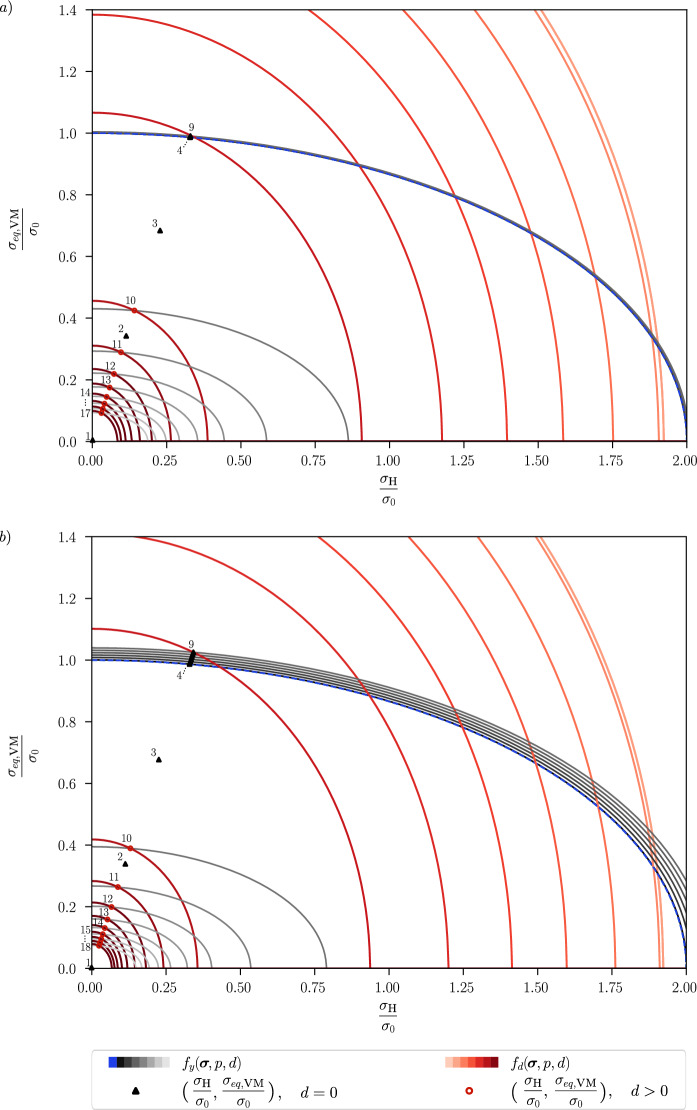
Stress evolution for a given strain path with the corresponding plastic yield surface $f_y(\varvec{\sigma },p,d)$, where the initial plastic yield surface $f_y(\varvec{\sigma },0,0)$ is marked in blue, and damage yield surface $f_d(\varvec{\sigma },p,d)$ in the hydrostatic-deviatoric plane. The evolution of the stress and the yield surfaces is shown in **a** for a hardening parameter of $H=500\,\text {MPa}$ and in **b** for a hardening parameter of $H=5000\,\text {MPa}$. The numbers represent the sequence of stress evolution under the loading process

In the following, we analyse the stress evolution for the three-dimensional homogeneous case under uniaxial tension. The stress triaxiality is accordingly $\eta ^*=1/3$. The material constants are given by $E=210~\text {GPa}$, $\nu =0.3$, $M=0.5$ and $G_c=150~\text {N/mm}$. For a given constant strain increment, the stresses are computed numerically as described in Sect. 2.4. The resulting material response is shown in Fig. 4, where we illustrate the stress evolution in the hydrostatic-deviatoric plane for a monotonic loading and the corresponding plastic and damage yield surfaces $f_y(\varvec{\sigma }, p,d)$ and $f_d(\varvec{\sigma }, p,d)$.

Figure 4a) shows the material response for a hardening parameter $H=500\,\text {MPa}$, which is in the order of magnitude of the hardening parameter used for the simulations in Sect. 4. To better visualise the initial enlargement of the plastic yield surface during the plastic stage, the hardening parameter is increased to $H=5000\,\text {MPa}$ in Fig. 4b). The computed stress state is marked with the numbers next to it indicating the order in which these stresses occur during loading. The plastic yield surface $f_y(\varvec{\sigma }, p,d)$ first grows and then shrinks (corresponding to a development from black to white in Fig. 4), and the damage yield surface $f_d(\varvec{\sigma }, p,d)$ shrinks (development from orange to red) during the loading process.

As shown in Fig. 4, the stresses are initially located in the elastic domain, the corresponding initial yield surface is $f_y(\varvec{\sigma },0,0)$ (marked in blue). Plastic strain starts to evolve as soon as the plastic yield criterion is met, i.e. the stress is on the yield surface, and the yield surface then grows with increasing plastic strain. When both the plasticity and the damage criterion are met, i.e. $f_y(\varvec{\sigma },p,0)=0$,  $f_d(\varvec{\sigma },p,0)=0$, the stress state evolves along the intersection of the two shrinking yield surfaces. More precisely, damage and plasticity continue to evolve together, and the stress decreases. We notice a slow plastic evolution before the onset of damage, but a rapid decrease in stresses as soon as damage and plasticity evolve together, which slows down with a higher value of the phase-field variable *d*.

## Numerical simulations

Simulation results for different geometries are presented using the algorithm described in Sect. 2.4. In the following simulations, the Young’s modulus is set to $E=210~\text {GPa}$ and the Poisson’s ratio to $\nu =0.3$. The plastic parameters typical for steel and the fracture toughness $G_c$ are specified individually for the geometries under consideration.

### Axisymmetric specimen under tension

We first consider an axisymmetric specimen under tension. The specimen is clamped at one end and subjected to a uniform displacement  $\bar{\varvec{u}}=(U,0,0)$ at the other end. The overall length is set to $L_0=1~\text {mm}$, and the internal length to $\ell =0.15~\text {mm}$. The specimen is discretised by four-node bilinear axisymmetric elements with a uniform mesh size of $h_e=5\cdot 10^{-3}~\text {mm}$, which provides a sufficiently high resolution to display a smooth distribution of the phase-field variable and of the cumulated plastic strain over the length of the bar. The specimen has a small imperfection, namely, the diameter of the cross-section is decreased by $2\cdot 10^{-4}~\text {mm}$ in the centre and gradually increases to the full diameter of $D_0=0.1~\text {mm}$ over a total length of $0.2~\text {mm}$. The element size is adjusted accordingly in the neighbourhood of the small defect. The plastic parameters are defined by an initial yield stress of $\sigma _0=730~\text {MPa}$, a hydrostatic parameter $M=0.5$ and a hardening parameter $H=500~\text {MPa}$, and the fracture toughness is given by $G_c=10~\text {N/mm}$.

**Fig. 5:**
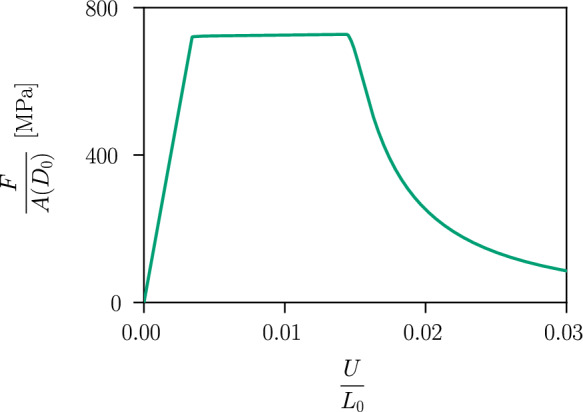
Stress–strain response of the axisymmetric specimen for $G_c=10~\text {N/mm}$

**Fig. 6:**
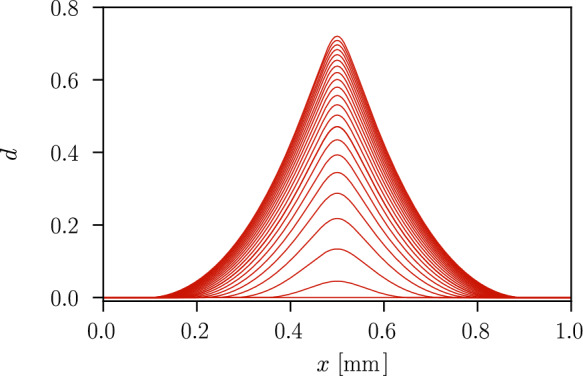
Evolution of the distribution of the phase-field variable *d* over the length of the axisymmetric specimen along the symmetry axis

**Fig. 7:**
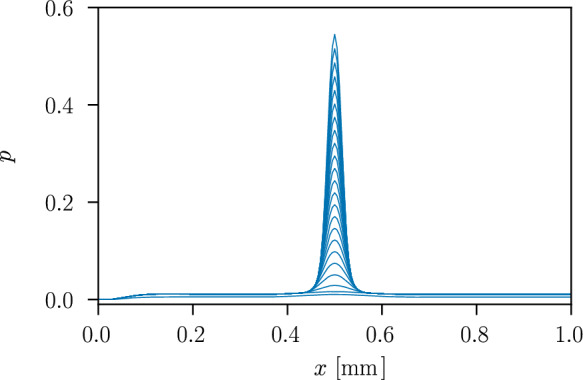
Evolution of the distribution of the cumulated plastic strain *p* over the length of the axisymmetric specimen along the symmetry axis

**Fig. 8:**
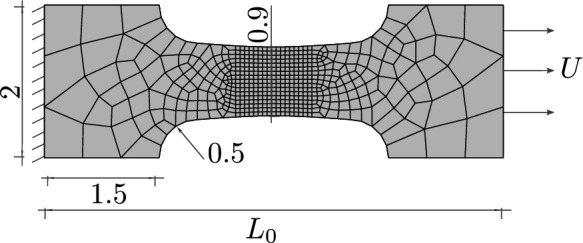
Geometry, boundary conditions and mesh of a plane strain specimen with an element size of $h_e=0.02~\text {mm}$ in the centre. For visualisation purposes, the mesh size is increased by a factor of three

The stress $\sigma =F/A(D_0)$ in the axisymmetric specimen depending on the evolution of the strain $\varepsilon =U/L_0$ is shown in Fig. 5, where *A* denotes the surface of the cross-section with a diameter of $D_0=0.1~\text {mm}$. The material response describes an elastic stage, a purely plastic stage with linear isotropic hardening and a softening stage, where the stress in the material decreases. The onset of softening at a strain of $\varepsilon =0.014$ and a stress of $\sigma =728.2~\text {MPa}$ coincides with the onset of damage, i.e. $d>0$, in the centre of the specimen. Compared to the one-dimensional example in Sect. 3.1, here localisation of the plastic strain occurs and a three-dimensional stress state arises where the effect of the hydrostatic stress leads to the onset of softening at a lower strain. The evolving phase-field profile over the length of the bar is illustrated in Fig. 6, showing the finite support of the phase-field variable. The damage is regularised over a damage zone proportional to $\ell$ [20, 58], while plasticity is strongly localised in the centre of the damage zone where the imperfection is located, as illustrated in Fig. 7.

### Two-dimensional plane strain specimens

Two differently shaped two-dimensional plane strain specimens are considered to investigate the damage initialisation and evolution as well as its connection to the deviatoric and hydrostatic stress. The specimens are discretised by four-node bilinear plane strain elements. The geometry of the first specimen, similar to a geometry used in [60], is shown in Fig. 8. The specimen with a length of $L_0=6~\text {mm}$ is clamped on the left-hand side, and a uniform displacement *U* is applied on the right-hand side. The mesh is refined in the centre, where plastic strain and damage are expected to arise, with a uniform mesh size of $h_e=0.02~\text {mm}$. The internal length of the phase-field model is set to $\ell =0.15~\text {mm}$. The material parameters are $\sigma _0=730~\text {MPa}$, $M=0.5$, $H=200~\text {MPa}$, and the fracture toughness is $G_c=30~\text {N/mm}$.

**Fig. 9:**
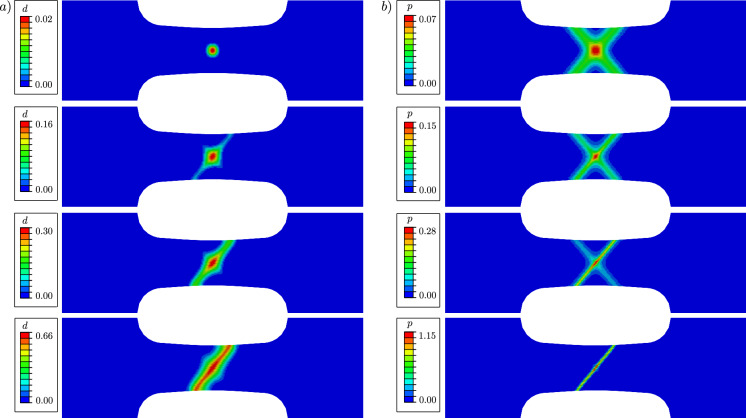
**a** Evolution of the phase-field variable *d* with the average strain $\varepsilon =U/L_0$, compared to **b** the evolution of the cumulated plastic strain *p*. The images in the same row are related to the same average strain of $\varepsilon =0.006$, $\varepsilon =0.007$, $\varepsilon =0.008$ and $\varepsilon =0.012$, respectively

**Fig. 10:**
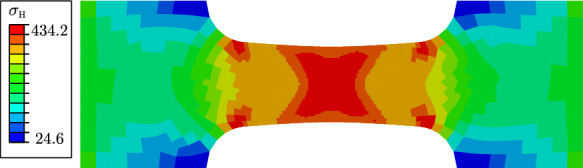
Distribution of the hydrostatic stress $\sigma _\textsc {h}$ at an average strain of $\varepsilon =0.004$. The hydrostatic stress concentrates in the centre, where damage formation is triggered

**Fig. 11:**
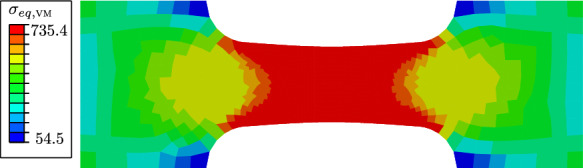
Distribution of the deviatoric part of the stress tensor represented by the von Mises equivalent stress $\sigma _{eq,\textsc {vm}}$ at an average strain of $\varepsilon =0.004$

Figure 9 illustrates the evolution of the phase-field variable *d* and the cumulated plastic strain *p* at different average strain levels $\varepsilon =U/L_0$. Damage occurs first in the centre of the specimen, where the hydrostatic stress $\sigma _\textsc {h}$ concentrates, see Fig. 10. As shown in Fig. 11, the deviatoric part of the equivalent stress in (4), represented by $\sigma _{eq,\textsc {vm}}$, is distributed over the whole gauge area. The cumulated plastic strain *p* in Fig. 9b) concentrates in shear bands crossing in the centre of the specimen. The phase-field variable continues to evolve in one of the shear bands, in which *p* concentrates and which becomes predominant over the other.

**Fig. 12:**
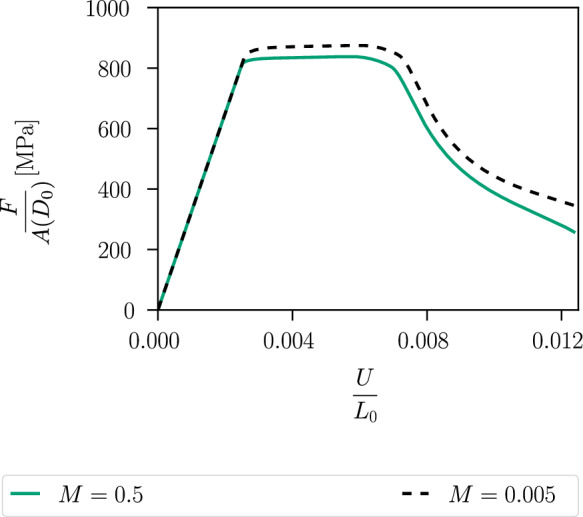
Global material response of the specimen shown in Fig. 8 for two different values of *M*

**Fig. 13:**
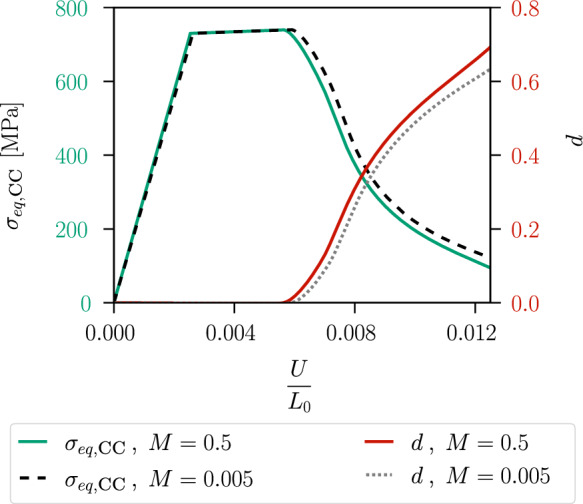
Local response of one critical element in the specimen centre. The equivalent stress $\sigma _{eq,\textsc {cc}}$ and the phase-field variable *d* are shown versus the average strain for two different values of *M*

The global behaviour of the specimen is shown in Fig. 12, where the nominal stress is expressed as a function of the average strain. The nominal stress is given by $\sigma =F/A(D_0)$, where *A* denotes the surface of the centre cross-section with a width of $D_0=0.9~\text {mm}$ and a thickness of $1~\text {mm}$. We compare two curves corresponding to two different values of the hydrostatic parameter *M*. For a smaller *M*, a higher deviatoric stress $\varvec{\sigma }_{dev}$ is required to fulfil the yield criterion, resulting in a higher overall stress level. For a critical element in the centre of the specimen, the local phase-field evolution and the local equivalent stress $\sigma _{eq,\textsc {cc}}$ are illustrated in Fig. 13, showing that the onset of stress softening coincides with the onset of phase-field evolution. The equivalent stress in the purely plastic stage is independent of the choice of the parameter *M* and is equal to the yield stress $\sigma _y$. However, for a smaller *M* the onset of damage begins at a larger global strain, which also affects the evolution of the equivalent stress $\sigma _{eq,\textsc {cc}}$.

**Fig. 14:**
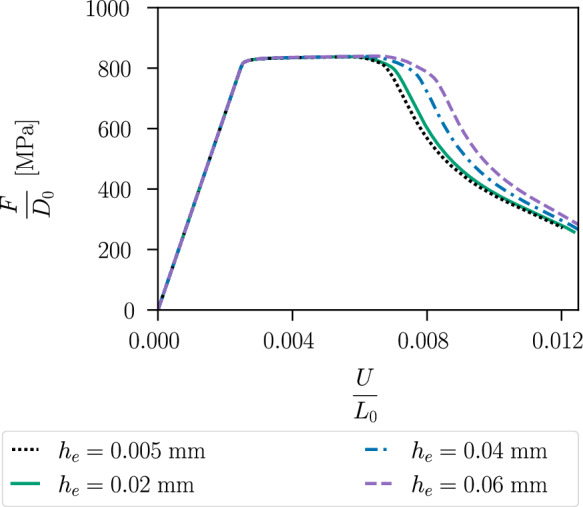
Global stress–strain curves of the specimen shown in Fig. 8 for different mesh refinements with corresponding element size $h_e$

Although the plastic strain is localised in shear bands, the damage-plasticity problem is well-posed, as the damage is regularised by the phase-field. Even in the plastic limit case, when the plastic strain becomes a bounded Radon measure, the discretised measure is mesh-independent [61, 20]. In Fig. 14, the mesh independency of the proposed model is verified. For this purpose, different mesh refinements in the centre of the specimen are considered, more precisely $h_e=0.005\,\text {mm}$, $h_e=0.02\,\text {mm}$, $h_e=0.04\,\text {mm}$, $h_e=0.06\,\text {mm}$, where the internal length remains fixed at $\ell =0.15\,\text {mm}$. The material response, i.e. the solutions for the displacement and the phase-field variable, converges with mesh refinement towards the exact solution, which is best approximated by the finest mesh, here given by $h_e = 0.005 \,\text {mm}$, which indicates mesh independency.

**Fig. 15:**
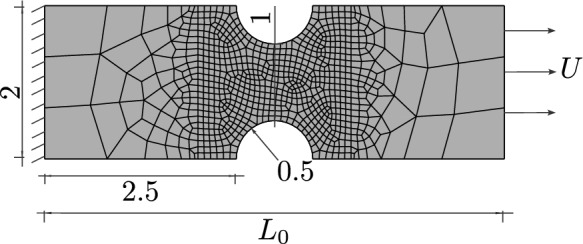
Geometry, boundary conditions and mesh of a notched plane strain specimen. The specimen has two notches with a radius of $0.5~\text {mm}$ and a clamped boundary on the left. The mesh size is set to $h_e=0.02~\text {mm}$ in the centre. For visualisation purposes, the mesh size is increased by a factor of four

**Fig. 16:**
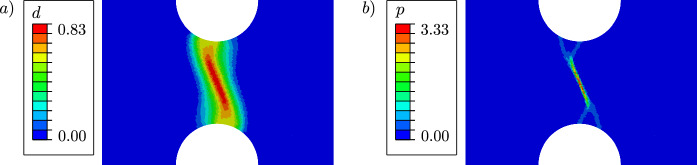
Distribution of **a** the phase-field variable *d* and **b** the cumulated plastic strain *p* at an average strain of $\varepsilon =0.012$

Another plane strain specimen with pre-defined notches in the centre is shown in Fig. 15. The initial length is set to $L_0=6~\text {mm}$, and the constitutive parameters, length scale parameter, element type, and mesh size are the same as in the previous example. The specimen shows a similar ductile fracture behaviour as the one discussed above. Figure 16 illustrates the distribution of the phase-field variable *d* and the cumulated plastic strain *p* at an average strain of $\varepsilon =0.012$. As illustrated in Fig. 16a), the maximum value of the phase-field variable is reached in the centre, where a non-straight regularised crack path can be observed between the two notches. The cumulated plastic strain *p* in Fig. 16b) localises in the centre and splits into two branches near the notches, while the phase-field variable concentrates in one of the branches. As illustrated in Fig. 16, damage is regularised by the phase-field approach, however, the plastic strain can accumulate locally and reach large values in the shear bands.

### Axisymmetric notched specimens under tension

**Fig. 17:**
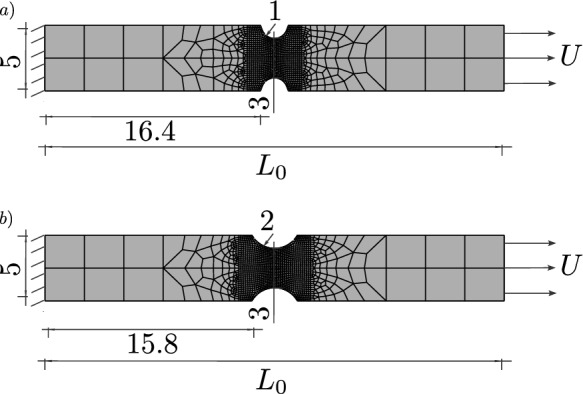
Geometry, boundary conditions and mesh, mirrored on the symmetry axis, of an axisymmetric specimen with **a** notch radius of $1~\text {mm}$, **b** notch radius of $2~\text {mm}$ and a diameter of $5~\text {mm}$, reduced to $3~\text {mm}$ in the centre. For visualisation purposes, the mesh size is increased by a factor of three

In this section, we apply the proposed phase-field model to two axisymmetric specimens with a different notch radius at the centre to demonstrate the influence of the stress triaxiality, i.e. the hydrostatic stresses, on the ductile fracture behaviour. The specimen with a notch radius of $1~\text {mm}$ is shown in Fig. 17a) and the specimen with a notch radius of $2~\text {mm}$ in Fig. 17b). The boundary on the left-hand side is clamped, while the specimens are subjected to a uniform displacement *U* on the right-hand side. The total length is given by $L_0=35~\text {mm}$ with a minimum diameter in the centre of $3~\text {mm}$. The specimens are discretised by four-node bilinear axisymmetric elements. The mesh size in the centre is set to $h_e=0.05~\text {mm}$, and the internal length to $\ell =0.15~\text {mm}$. The hydrostatic parameter is defined by $M=0.8$ to ensure a significant influence of the hydrostatic stress. The initial yield stress is given by $\sigma _0=730~\text {MPa}$, the hardening parameter by $H=200~\text {MPa}$, and the fracture toughness is set to $G_c=300~\text {N/mm}$.

**Fig. 18:**
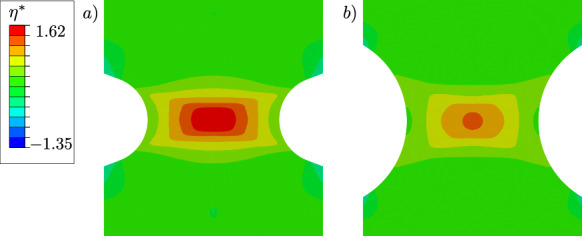
Stress triaxiality $\eta ^*$ for the specimen with **a** notch radius of $1\,\text {mm}$, **b** notch radius of $2\,\text {mm}$ at an average strain of $\varepsilon =0.0025$

With this example, we aim at showing that the proposed variational model can account for varying stress triaxialities that occur in the differently notched specimens. The stress triaxiality $\eta ^*=\sigma _{\textsc {h}}/\sigma _{eq,\textsc {vm}}$ for the two specimens is shown in Fig. 18 at an average strain $\varepsilon =U/L_0=0.0025$. This strain corresponds to the plastic stage before the phase-field variable starts to evolve. In the critical element in the centre, the specimen with a notch radius of $1~\text {mm}$ in Fig. 18a) shows a stress triaxiality of $\eta ^*=1.62$, whereas the specimen with the larger notch radius of $2~\text {mm}$ shows a lower stress triaxiality of $\eta ^*=1.52$. The inhomogeneous distribution of the hydrostatic stress has an influence on the location of the crack initiation [62], resulting in fracture initiation in the centre of the specimens, and the amount of stress triaxiality is expected to have an influence on the global stress–strain curve, in particular on the onset of material softening.

**Fig. 19:**
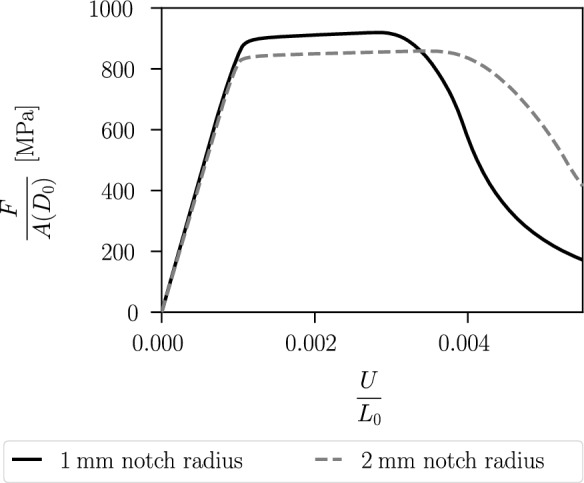
Global stress–strain curve for the specimen with notch radius of $1\,\text {mm}$ (solid black) and with notch radius of $2\,\text {mm}$ (dashed gray)

**Fig. 20:**
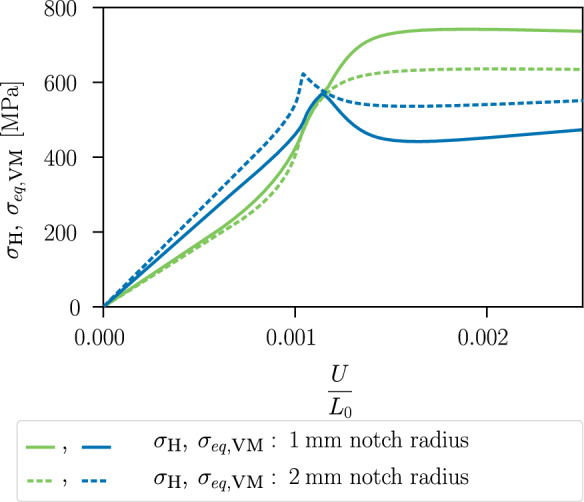
Local evolution of the hydrostatic stress $\sigma _\textsc {h}$ and the von Mises equivalent stress $\sigma _{eq,\textsc {vm}}$ in the critical element versus the average strain for the specimen with notch radius of $1\,\text {mm}$ (solid line) and with notch radius of $2\,\text {mm}$ (dashed line)

Figure 19 shows the stress–strain curves. The specimen with a notch radius of $1~\text {mm}$ shows an earlier material softening than the one with a notch radius of $2~\text {mm}$. This is due to the incorporation of the hydrostatic stress in the proposed model coupling plasticity and the phase-field variable, which thus takes into account the different stress triaxiality of the two specimens for modelling fracture initiation. Figure 20 illustrates the local hydrostatic stress $\sigma _\textsc {h}$ and the von Mises equivalent stress $\sigma _{eq,\textsc {vm}}$ in the critical element in the centre of both specimens during the purely elastic and plastic stage before damage initiation. Compared to the specimen with notch radius of $2~\text {mm}$, the specimen with notch radius of $1~\text {mm}$ shows a higher hydrostatic stress $\sigma _\textsc {h}$ and a lower von Mises equivalent stress $\sigma _{eq,\textsc {vm}}$.

**Fig. 21:**
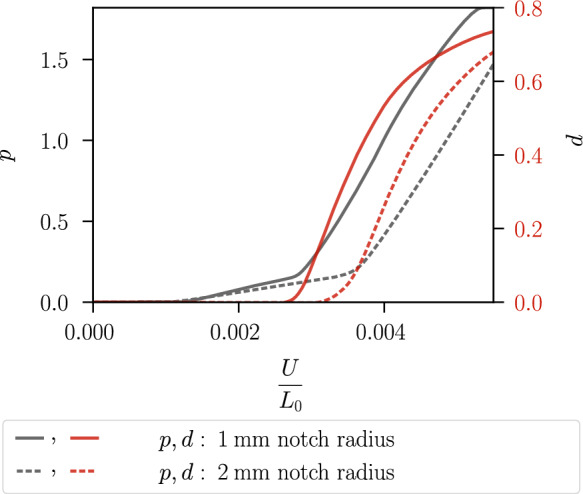
Local evolution of the cumulated plastic strain *p* and the phase-field *d* versus the average strain in the critical element for the specimen with notch radius of $1\,\text {mm}$ (solid line) and with notch radius of $2\,\text {mm}$ (dashed line)

**Fig. 22:**
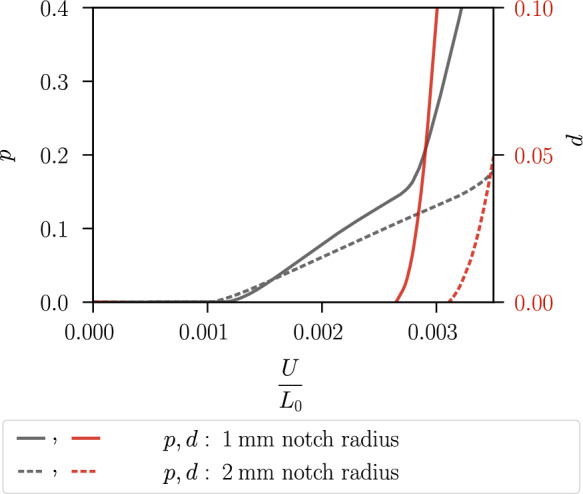
Detail of Fig. 21 illustrating the purely elastic and plastic stage until the onset of damage

The reason for the different fracture initiation and thus material softening of the two specimens is analysed in Figs. 21 and 22. The cumulated plastic strain *p* and the phase-field *d* in the critical element in the centre of both specimens are shown in Fig. 21. The kink in the evolution of the cumulated plastic strain occurs because, as the phase-field develops, plasticity becomes localised in two rows of elements where a significant increase in plastic strain is observed. The phase-field starts to evolve in the two specimens at a different strain; in the critical element of the specimen with a notch radius of $1~\text {mm}$ at a lower average strain of $U/L_0=0.0026$, in the critical element of the other specimen at an average strain of $U/L_0=0.0031$. Figure 22 shows a detail of Fig. 21 considering the strain interval $U/L_0=[0,0.0035]$, as this shows the different evolution of *p* in the specimens that triggers the damage initiation. The phase-field *d* starts to develop in both specimens when the cumulated plastic strain reaches the value $p=0.14$. Thus, the different growth of the cumulated plastic strain in the purely plastic stage leads to damage initiation at a different average strain.

The specific choice of *M* plays an important role in weighting the hydrostatic stress or strain against the deviatoric components. It can be observed that a smaller $0\le M<1$ has a greater influence on the hydrostatic part of the plastic strain tensor in (8), while reducing the influence on the equivalent stress $\sigma _{eq,\textsc {cc}}(\varvec{\sigma })$ defined in (4) at the same time. Since *M* determines the influence of the hydrostatic stress on the equivalent stress during plastic deformation, it is the parameter that, in addition to the hydrostatic stress arising in the elastic stage, causes a different stress–strain behaviour with different triaxiality $\eta ^*$. For *M* close to zero, the hydrostatic stress hardly influences the equivalent stress $\sigma _{eq,\textsc {cc}}$ during plastic yielding. In this case, the different stress levels visible in the stress–strain curves of the two specimens are only due to triaxiality effects in the elastic stage caused by the predefined notches, resulting in different hydrostatic stresses and volumetric elastic strains. However, ductile fracture initiation cannot be influenced by triaxiality effects for *M* close to zero.

**Fig. 23:**
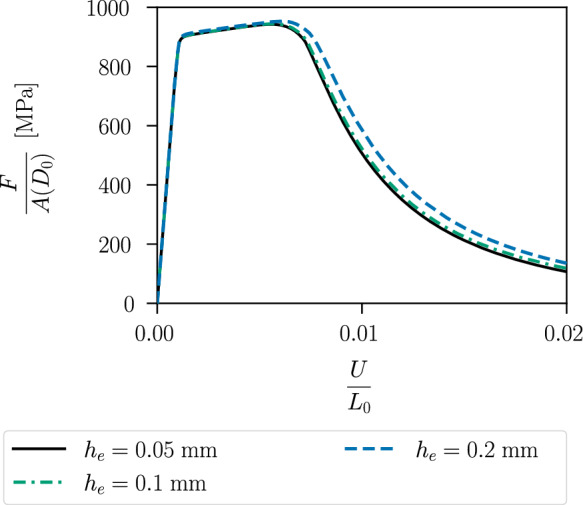
Stress–strain curves of the specimen with notch radius of $1\,{\text{mm}}$ for different mesh refinements with corresponding element size $h_e$

Figure 23 shows the stress–strain curves of the geometry with a notch radius of $1\,\text {mm}$ for different mesh sizes. The mesh size in the centre is set to $h_e = 0.05\,\text {mm}$, $h_e = 0.1\, \text {mm}$ or $h_e = 0.2\,\text {mm}$ with an internal length of $\ell = 0.3\,\text {mm}$, so that $h_e < \ell$ applies to all three simulations. The stress–strain curves underline again the mesh independency of the proposed model, see also Sect. 4.2.

## Conclusion and perspectives

This paper demonstrates the potential of using the modified Cam-Clay plasticity model originating from soil mechanics to describe ductile material behaviour, for example of metals. The underlying physical processes, such as the formation of voids in metals, lead to ductile fracture which depends on triaxiality effects, in turn due to a three-dimensional stress state resulting from necking or notches. In this context, the magnitude and distribution of the hydrostatic stress is crucial for the fracture initiation. The yield surface of the modified Cam-Clay model is an ellipse along the hydrostatic axis in the principal stress space instead of a cylinder as for von Mises plasticity, so that the volumetric part of the stress and strain tensor is considered in addition to the deviatoric part during plastic deformation. This work represents a step towards a variationally consistent formulation that accounts for the triaxiality effects on ductile fracture initiation by coupling modified Cam-Clay plasticity with a regularising phase-field model and ensures mesh independence.

We have shown that the proposed coupled model can reproduce the ductile fracture behaviour with its typical successive occurrence of elastic, plastic and softening phases through analytical calculations and various numerical simulations. The triaxiality effects are captured qualitatively so that the fracture initiation at different global strains can be modelled for geometries with different stress triaxiality. However, the model shows limited flexibility to adapt the magnitude of the triaxiality effects to the range of possible material responses. Therefore, it is necessary to further increase the influence of the stress triaxiality beyond the adjustment of the parameter *M*.

From the numerical perspective, an improvement of the convergence behaviour of the staggered algorithm using the implicit solver is desirable. This behaviour is currently sensitive to many interacting factors, such as the Newton–Raphson iterations in the plasticity return map, the penalty term, the loading process, the fracture toughness, the geometry and the chosen (pseudo) time step. Furthermore, we suggest to add geometric nonlinearity to the proposed model in order to predict the ductile fracture behaviour in real experiments.

To conclude, the limitations of the model are emphasised. The limited adaptability of the model to different material responses due to only one parameter, which enters the formulation through the yield surface and adjusts the effect of hydrostatic stresses and thus the onset of material softening, demands further work in this direction. In addition, a comparison of the simulation results with experimental tests for ductile fracture requires the extension of the variational model to large strains.

## Appendix: A. Dual norm for the Cam-Clay criterion

We show as follows how to obtain the expression (6) for the dual strain norm $\Vert {\dot{{\varvec{\varepsilon }}}^p}\Vert _{\textsc {m}}^*$ of the Cam-Clay stress norm $\Vert \varvec{\sigma }\Vert _{\textsc {m}}$. The following minimisation problem defines the dual norm

49 $$\begin{aligned} \Vert \dot{{{\varvec{\varepsilon }}}}^p\Vert _{\textsc {m}}^*:=\sup _{\Vert \varvec{\sigma }\Vert _{\textsc {m}}\le \sigma _y}\frac{\varvec{\sigma }}{\sigma _y(p)}\cdot {\dot{{\varvec{\varepsilon }}}^p}\quad =\sup _{\Vert \tilde{\varvec{\sigma }}\Vert _{\textsc {m}}\le 1}\tilde{\varvec{\sigma }}\cdot {\dot{{\varvec{\varepsilon }}}^p}\quad \text {with}\quad \Vert \tilde{\varvec{\sigma }}\Vert _{\textsc {m}}:=\sqrt{{\frac{3}{2}\,\Vert \tilde{\varvec{\sigma }}_{dev}\Vert ^2+M^2\,\tilde{\sigma }_{\textsc {h}}^2}} \end{aligned}$$

and $\tilde{\varvec{\sigma }}:=\varvec{\sigma }/\sigma _y$. Using the Lagrange multiplier method, the solution of the optimisation problem (7) including the convex constraint $\Vert \tilde{\varvec{\sigma }}\Vert _{\textsc {m}}\le 1$ must be a stationary point of the Lagrangian

50 $$\begin{aligned} \mathcal {L}(\tilde{\varvec{\sigma }}_{dev},\sigma _\textsc {h},\tilde{\lambda })= \tilde{\varvec{\sigma }}_{dev}\cdot {{\dot{{\varvec{\varepsilon }}}^p_{dev}}}+ \tilde{\sigma }_\textsc {h}\;\text{tr}({\dot{{\varvec{\varepsilon }}}^p}) +\tilde{\lambda }\left[ 1- \left[ {\frac{3}{2}\,\Vert \tilde{\varvec{\sigma }}_{dev}\Vert ^2+M^2\,\tilde{\sigma }_{\textsc {h}}^2}\right] \right] \, \end{aligned}$$

for any deviatoric tensor $\tilde{\varvec{\sigma }}_{dev}$, scalar $\tilde{\sigma }_\textsc {h}$, and Lagrange multiplier $\tilde{\lambda }\ge 0$ [63]. The first-order optimality conditions with respect to $\tilde{\varvec{\sigma }}_{dev}$ and $\tilde{\sigma }_\textsc {h}$ give

51 $$\begin{aligned} {\dot{{\varvec{\varepsilon }}}^p_{dev}}-3\,\tilde{\lambda }\,\tilde{\varvec{\sigma }}_{dev}=\textbf{0}\,,\qquad \text{tr}({\dot{{\varvec{\varepsilon }}}^p})-2M^2\,\tilde{\lambda }\,\tilde{\sigma }_\textsc {h}=0\,, \end{aligned}$$

which imply in particular that $\tilde{\varvec{\sigma }}_{dev}$ is co-axial to $\dot{{\varvec{\varepsilon }}}^p_{dev}$.

The unilateral optimality condition with respect to the non-negative Lagrange multiplier is in the form of a set of Karush-Kuhn-Tucker complementarity conditions [63]

52 $$\begin{aligned} \text {(i) }\;\tilde{\lambda }\ge 0\,,\qquad \text {(ii) }\; 1- \left[ \frac{3}{2}\,\Vert \tilde{\varvec{\sigma }}_{dev}\Vert ^2+M^2\,\tilde{\sigma }_{\textsc {h}}^2\right] \ge 0\,,\qquad \text {(iii) }\; \left[ 1- \left[ \frac{3}{2}\,\Vert \tilde{\varvec{\sigma }}_{dev}\Vert ^2+M^2\,\tilde{\sigma }_{\textsc {h}}^2\right] \right] \tilde{\lambda }=0\,. \end{aligned}$$

The case $\tilde{\lambda }=0$ corresponds to the elastic regime in which ${\dot{{\varvec{\varepsilon }}}^p_{dev}}=\textbf{0}$ and $\text{tr}({\dot{{\varvec{\varepsilon }}}^p})=0$. In the plastic regime $\tilde{\lambda }>0$ and the unilateral constraint (52ii) must be satisfied as an equality. Solving the optimality conditions (51) for the stress and replacing $\tilde{\varvec{\sigma }}_{dev}$, $\tilde{\sigma }_\textsc {h}$ in the equality constraint gives the solution for the Lagrange multiplier

$$\begin{aligned} \tilde{\lambda }= \frac{1}{2}\sqrt{\frac{2}{3}\,\Vert {\dot{{\varvec{\varepsilon }}}^p_{dev}}\Vert ^2+ \frac{\text{tr}^2({\dot{{\varvec{\varepsilon }}}^p})}{M^2}}. \end{aligned}$$

Finally, replacing the optimal stresses from (51) in the expression for the dual norm in (49) gives the result

$$\begin{aligned} \Vert {\dot{{\varvec{\varepsilon}}}^p}\Vert _{\textsc {m}}^*=\sqrt{\frac{2}{3}\,\Vert {\dot{{\varvec{\varepsilon }}}^p_{dev}}\Vert ^2+ \frac{\text{tr}^2({\dot{{\varvec{\varepsilon }}}^p})}{M^2}}. \end{aligned}$$

One can similarly prove that

53 $$\begin{aligned} \Vert \tilde{\varvec{\sigma }}\Vert _{\textsc {m}}^{**}:= \sup _{\Vert {\dot{{\varvec{\varepsilon}}}^p}\Vert _{\textsc {m}}^{*}\le 1} \tilde{\varvec{\sigma }}\cdot {\dot{{\varvec{\varepsilon }}}^p}=\Vert \tilde{\varvec{\sigma }}\Vert _{\textsc {m}}. \end{aligned}$$

## B. Variational derivation of the strong form plasticity equations and return mapping algorithm

We derive here the strong optimality conditions for the minimisation problem (12) of the elasto-plastic energy functional (13). Subsequently, we formulate a return mapping algorithm for time integration. The extension to the case including the phase-field damage variable is classical and it is not reproduced here. It can be found for example in [64] for the case without plasticity and in [18, 19] for the case with coupled damage and plasticity.

### Strong form equations

The solution $(\varvec{u},{\varvec{\varepsilon }}^p)\in \mathcal {V}_i\times \mathcal {P}$ of the problem (12) must verify the condition of unilateral local directional minimality, i.e. for all test functions $(\varvec{v},\varvec{\eta })\in H_0^1(\Omega ,\mathbb {R}^3)\times L^2(\Omega ,\mathbb {R}^{3,3})$, there exists $\bar{h}>0$ such that for all $h\in (0,\bar{h})$

54 $$\begin{aligned} \mathcal {E}_i(\varvec{u}+h\varvec{v},{\varvec{\varepsilon }}^p+h\varvec{\eta })-\mathcal {E}_i(\varvec{u},{\varvec{\varepsilon }}^p)\ge 0\,, \end{aligned}$$

where $\mathcal {V}_i$ and $\mathcal {P}$ are the space of admissible displacements at the *i*-th time step and the space of admissible plastic strain increments, respectively.

Taking $\varvec{\eta }=\textbf{0}$ in (54) gives the standard variational formulation of the elastic sub-problem, which delivers the equilibrium equations for the stress tensor $\varvec{\sigma }$ along with the associated Neumann boundary conditions, see (15). The formal proof is classical and it is not reproduced here.

The case with $\varvec{v}=\textbf{0}$ and $\varvec{\eta }\ne \textbf{0}$ is less standard, because the presence of the norm function renders the energy non-smooth with respect to ${\varvec{\varepsilon }}^p$. The energy increment in this case is given by

$$\begin{aligned} \mathcal {E}_i(\varvec{u},{\varvec{\varepsilon }}^p&+h\varvec{\eta })-\mathcal {E}_i(\varvec{u},{\varvec{\varepsilon }}^p)\\= & \int _\Omega h\,\frac{\partial \psi _e}{\partial {\varvec{\varepsilon }}^p}({\varvec{\varepsilon }}(\varvec{u})-{\varvec{\varepsilon }}^p)\cdot \,\varvec{\eta }\,\text{d}\varvec{x}\\ & +\int _\Omega \left[ \psi _p\left( p_{i-1} + \Vert \Delta {\varvec{\varepsilon }}^p+h\,\varvec{\eta }\Vert _{\textsc {m}}^*\right) -\psi _p\left( p_{i-1}+ \Vert \Delta {\varvec{\varepsilon }}^p\Vert _{\textsc {m}}^* \right) \right] \,\text{d}\varvec{x}+o(h)\\= & \int _\Omega -h\,\varvec{\sigma }({\varvec{\varepsilon }}(\varvec{u})-{\varvec{\varepsilon }}^p)\cdot \varvec{\eta }\,\text{d}\varvec{x}\\ & +\int _\Omega \left[ \psi _p\left( p_{i-1} + \Vert \Delta {\varvec{\varepsilon }}^p+h\,\varvec{\eta }\Vert _{\textsc {m}}^*\right) -\psi _p\left( p_{i-1}+ \Vert \Delta {\varvec{\varepsilon }}^p\Vert _{\textsc {m}}^* \right) \right] \,\text{d}\varvec{x}+o(h) \end{aligned}$$

where $\Delta {\varvec{\varepsilon }}^p={\varvec{\varepsilon }}^p-{\varvec{\varepsilon }}^p_{i-1}$ is the plastic strain increment. In the expression above, we expand the derivative of the first term exploiting the smoothness of the elastic energy density with respect to the elastic strain and that

$$\varvec{\sigma }=-\frac{\partial \psi _e}{\partial {\varvec{\varepsilon }}^p}({\varvec{\varepsilon }}-{\varvec{\varepsilon }}^p)= K\,\text{tr}({\varvec{\varepsilon }}-{\varvec{\varepsilon }}^p)\,\varvec{I}_3 + 2\mu \,\left[ {\varvec{\varepsilon }}_{dev}-{\varvec{\varepsilon }}_{dev}^p\right] .$$

For the second term, we should separately consider the case of solution with $\Vert \Delta {\varvec{\varepsilon }}^p\Vert _{\textsc {m}}^*$ equal to zero or not, as follows.

- If $$\Vert \Delta {\varvec{\varepsilon }}^p\Vert _{\textsc {m}}^*=0$$,
  $$\begin{aligned} \mathcal {E}_i(\varvec{u},{\varvec{\varepsilon }}^p&+h\varvec{\eta })-\mathcal {E}_i(\varvec{u},{\varvec{\varepsilon }}^p)\\= &\,\, h \int _\Omega \left[ -\,\varvec{\sigma }\cdot \varvec{\eta }+\psi _p'(p_{i-1})\,\Vert \varvec{\eta }\Vert _{\textsc {m}}^* \right] \,\text{d}\varvec{x}+o(h)\\= & \,\,h \int _\Omega \left[ -\,\varvec{\sigma }\cdot \varvec{\eta }+\sigma _y(p_{i-1})\,\Vert \varvec{\eta }\Vert _{\textsc {m}}^* \right] \,\text{d}\varvec{x}+o(h) \end{aligned}$$
  where we used $\psi _p'(p_{i-1})=\sigma _y(p_{i-1})=\sigma _0+H \,p_{i-1}$ (note also that in this case $p=p_{i-1}$). Taking $\varvec{\eta }(\varvec{x})=\tilde{\varvec{\eta }}\,\eta (\varvec{x})$, where $\eta \in L^2(\Omega )$ denotes an arbitrary scalar function and $\tilde{\varvec{\eta }}\in \mathbb {R}^{3,3}$ an arbritrary constant, yields the pointwise necessary optimality condition
  $$\varvec{\sigma }\cdot \tilde{\varvec{\eta }}\le \sigma _y(p)\,\Vert \tilde{\varvec{\eta }}\Vert _{\textsc {m}}^*\quad \text {in}\quad \Omega$$
  for all $\tilde{\varvec{\eta }}\in \mathbb {R}^{3,3}$. In particular
  $$\Vert \varvec{\sigma }\Vert _{\textsc {m}}^{**}:=\sup _{\Vert {\tilde{\varvec{\eta }}}\Vert _{\textsc {m}}^*\le 1}\varvec{\sigma }\cdot \tilde{\varvec{\eta }}\le \sigma _y(p)\,,$$
  where $$\Vert \varvec{\sigma }\Vert _{\textsc {m}}^{**}=\Vert \varvec{\sigma }\Vert _{\textsc {m}}$$ is the dual of the dual Cam-Clay norm, coinciding with the Cam-Clay norm itself, see (53). Hence the optimality condition implies that
  $$f_y(\varvec{\sigma },p)=\Vert \varvec{\sigma }\Vert _{\textsc {m}}-\sigma _y(p)\le 0\,,$$
  where $\Vert \varvec{\sigma }\Vert _{\textsc {m}}=\sigma _{eq,\textsc {cc}}(\varvec{\sigma })$ according to (6).
- If $\Vert \Delta {\varvec{\varepsilon }}^p\Vert _{\textsc {m}}^*>0$, the energy increment is given by
  $$\begin{aligned} &\mathcal {E}_i(\varvec{u},{\varvec{\varepsilon }}^p+h\varvec{\eta })-\mathcal {E}_i(\varvec{u},{\varvec{\varepsilon }}^p)\\ & = \int _\Omega \left[ -h\,\varvec{\sigma }\cdot \varvec{\eta } \right. \\ & \left.  \quad \quad \quad \!+\psi _p^\prime \left( p\right) \,\left( \Vert \Delta {\varvec{\varepsilon }}^p+h\,\varvec{\eta }\Vert _{\textsc {m}}^*-\Vert \Delta {\varvec{\varepsilon }}^p\Vert _{\textsc {m}}^*\right) \right] \,\text{d}\varvec{x}+o(h)\\ & = \,\,h \int _\Omega \left[ -\,\varvec{\sigma }\cdot \varvec{\eta }+\sigma _y(p)\, \frac{\partial \Vert \Delta {\varvec{\varepsilon }}^p\Vert _{\textsc {m}}^* }{\partial \Delta {{\varvec{\varepsilon }}}^p}\cdot {\varvec{\eta }}\right] \,\text{d}\varvec{x}+o(h) \end{aligned}$$
  and the arbitrariness of $\varvec{\eta }$ implies the necessary optimality condition
  55 $$\begin{aligned} \varvec{\sigma }= {\sigma _y(p)}\frac{\partial \Vert \Delta {\varvec{\varepsilon }}^p\Vert _{\textsc {m}}^* }{\partial \Delta {{\varvec{\varepsilon }}}^p}. \end{aligned}$$
  Since the derivative of the norm function reads
  $$\frac{\partial \Vert \Delta {\varvec{\varepsilon }}^p\Vert _{\textsc {m}}^* }{\partial \Delta {{\varvec{\varepsilon }}}^p} = \frac{\partial }{\partial \Delta {{\varvec{\varepsilon }}}^p}\, \sqrt{\frac{2}{3}\,\Vert \Delta {{\varvec{\varepsilon }}}^p_{dev}\Vert ^2+ \frac{\text{tr}(\Delta {{\varvec{\varepsilon }}}^p)^2}{M^2}} = \frac{1}{\Vert \Delta {\varvec{\varepsilon }}^p\Vert _{\textsc {m}}^*} \left[ \frac{2}{3}\,\Delta {{\varvec{\varepsilon }}}^p_{dev}+ \frac{\text{tr}(\Delta {{\varvec{\varepsilon }}}^p)}{M^2}\varvec{I}_3 \right] ,$$
  from (55) we infer that $\varvec{\sigma }$ and $\Delta {\varvec{\varepsilon }}^p$ are co-axial. Also, by computing the Cam-Clay norm of $\varvec{\sigma }$ in (55) and using the previous expression for the derivative of the norm function, it is immediate to show that the Cam-Clay criterion is verified as an equality, $\Vert \varvec{\sigma }\Vert _{\textsc {m}}=\sigma _y(p)$. Moreover, the derivative
  $$\frac{\partial f_y(\varvec{\sigma },p) }{\partial \varvec{\sigma }} = \frac{\partial \Vert \varvec{\sigma }\Vert _{\textsc {m}} }{\partial \varvec{\sigma }} = \frac{\partial }{\partial {\varvec{\sigma }}}\, \sqrt{\frac{3}{2}\,\Vert \varvec{\sigma }_{dev}\Vert ^2+ \frac{M^2\text{tr}^2({\varvec{\sigma }})}{9}} = \frac{1}{\Vert \varvec{\sigma }\Vert _{\textsc {m}}} \left[ \frac{3}{2}\,{\varvec{\sigma }}_{dev}+ \frac{M^2\text{tr}({\varvec{\sigma }})}{9}\varvec{I}_3 \right]$$
  is also co-axial to $\varvec{\sigma }$, hence to $\Delta {\varvec{\varepsilon }}^p$, and it is straightforward to show that
  56 $$\begin{aligned} \Delta {\varvec{\varepsilon }}^p=\Delta \lambda \frac{\partial f_y}{\partial \varvec{\sigma }} \end{aligned}$$
  with $\Delta \lambda :=\Vert \Delta {\varvec{\varepsilon }}^p\Vert ^*_{\textsc {m}}$, which is the incremental form of the classical normality rule of elasto-plasticity. For later convenience, let us define the director of the co-axial tensors as $\textbf{n}:=\varvec{\sigma }_{dev}/\Vert \varvec{\sigma }_{dev}\Vert$, so that it is also
  57 $$\begin{aligned} \Delta {\varvec{\varepsilon }}^p_{dev}=\Vert \Delta {\varvec{\varepsilon }}^p_{dev}\Vert \,\textbf{n} \end{aligned}$$
  In conclusion, the necessary condition for minimality of the total energy with respect to the plastic strain implies that
  $$f_y(\varvec{\sigma },p)=\Vert \varvec{\sigma }\Vert _{\textsc {m}}-\sigma _y(p)\le 0$$
  and
  58 $$\begin{aligned} {\left\{ \begin{array}{ll} \Delta {\varvec{\varepsilon }}^p=\textbf{0}\,,& \text {if}\quad \Vert \varvec{\sigma }\Vert _{\textsc {m}}<\sigma _y(p);\\ \Delta {\varvec{\varepsilon }}^p \text { is given by (56)}\,,& \text {if}\quad \Vert \varvec{\sigma }\Vert _{\textsc {m}}=\sigma _y(p). \end{array}\right. } \end{aligned}$$
  The conditions above can be also rewritten as follows
  59 $$\begin{aligned} \Delta {\varvec{\varepsilon }}^p=\Delta \lambda \,\frac{\partial f_y}{\partial {\varvec{\sigma }}},\quad \Delta \lambda \ge 0,\\f_y(\varvec{\sigma },p)\le 0,\quad f_y(\varvec{\sigma },p)\,\Delta \lambda =0, \end{aligned}$$
  which is the classical form of the Kuhn-Tucker conditions of elasto-plasticity.

### Return mapping algorithm

For integration of the incremental constitutive Eq. (59), it is useful to introduce the elastic predictor stress

60 $$\begin{aligned} \varvec{\sigma }^{tr}&:= K\,\text{tr}({\varvec{\varepsilon }}-{\varvec{\varepsilon }}^{p}_{i-1})\varvec{I}_3 + 2\,\mu \, [{\varvec{\varepsilon }}_{dev}-{\varvec{\varepsilon }}_{dev,i-1}^{p}]\\&\,=\varvec{\sigma }+ K \text{tr}(\Delta {\varvec{\varepsilon }}^p)\varvec{I}_3 + 2 \mu \Delta {\varvec{\varepsilon }}^p_{dev} \end{aligned}$$

which can be computed based on the current strain and on the quantities at the previous time step. The deviatoric component of the above equation yields

$$\varvec{\sigma }^{tr}_{dev}=\varvec{\sigma }_{dev} + 2 \mu \Delta {\varvec{\varepsilon }}^p_{dev}\,,$$

which shows that, since $\varvec{\sigma }_{dev}$ and $\Delta {\varvec{\varepsilon }}^p_{dev}$ are co-axial, also $\varvec{\sigma }^{tr}_{dev}$ must be, i.e.

61 $$\begin{aligned} \textbf{n}^{tr}:=\varvec{\sigma }^{tr}_{dev}/\Vert \varvec{\sigma }^{tr}_{dev}\Vert = \textbf{n}\,. \end{aligned}$$

Using (60), the optimality condition (55) can be written as

62 $$\begin{aligned} \varvec{\sigma }=\varvec{\sigma }^{tr}-{K}{\text{tr}(\Delta {{\varvec{\varepsilon }}}^p)}\varvec{I}_3-2\,\mu \, \Delta {\varvec{\varepsilon }}_{dev}^p= \left[ \frac{\sigma _y(p_{i-1})}{\Vert \Delta {\varvec{\varepsilon }}^p\Vert _{\textsc {m}}^*}+H\right] \left[ \frac{2}{3}\,\Delta {{\varvec{\varepsilon }}}^p_{dev}+ \frac{\text{tr}(\Delta {{\varvec{\varepsilon }}}^p)}{M^2}\varvec{I}_3 \right] \,, \end{aligned}$$

where we also used

$$\sigma _y(p)=\sigma _y(p_{i-1})+H \Delta p = \sigma _y(p_{i-1})+H \Vert \Delta {\varvec{\varepsilon }}^p\Vert ^*_{\textsc {m}}$$

stemming from (4). Taking the trace and the deviatoric part and recalling (57) and (61) results in

63 $$\begin{aligned} \left[ \frac{1}{M^2}\left[ \frac{\,\sigma _y(p_{i-1})}{\,\Vert \Delta {\varvec{\varepsilon }}^p\Vert _{\textsc {m}}^*}+H\right] +K \right] {\text{tr}(\Delta{\varepsilon^p)}}= & \frac{1}{3}\text{tr}(\varvec{\sigma }^{tr})\,, \end{aligned}$$

64 $$\begin{aligned} 2\left[ \frac{1}{3}\left[ \frac{\,\sigma _y(p_{i-1})}{\,\Vert\Delta{\varepsilon^p}\Vert _{\textsc {m}}^*}+H\right] +\,\mu \,\right] \Vert \Delta {{\varvec{\varepsilon }}}^p_{dev}\Vert= & \Vert \varvec{\sigma }^{tr}_{dev}\Vert \,, \end{aligned}$$

which is a system of two scalar nonlinear equations for $\text{tr}(\Delta {\varvec{\varepsilon }}^p)$ and $\Vert \Delta {\varvec{\varepsilon }}^p_{dev}\Vert$ given the elastic predictor.
